# Toward an Individual Binaural Loudness Model for Hearing Aid Fitting and Development

**DOI:** 10.3389/fpsyg.2021.634943

**Published:** 2021-06-22

**Authors:** Iko Pieper, Manfred Mauermann, Birger Kollmeier, Stephan D. Ewert

**Affiliations:** Medizinische Physik and Cluster of Excellence Hearing4All, Universität Oldenburg, Oldenburg, Germany

**Keywords:** loudness summation, hearing aid, hearing impairment, binaural inhibition, binaural summation, binaural loudness summation, loudness function

## Abstract

The individual loudness perception of a patient plays an important role in hearing aid satisfaction and use in daily life. Hearing aid fitting and development might benefit from individualized loudness models (ILMs), enabling better adaptation of the processing to individual needs. The central question is whether additional parameters are required for ILMs beyond non-linear cochlear gain loss and linear attenuation common to existing loudness models for the hearing impaired (HI). Here, loudness perception in eight normal hearing (NH) and eight HI listeners was measured in conditions ranging from monaural narrowband to binaural broadband, to systematically assess spectral and binaural loudness summation and their interdependence. A binaural summation stage was devised with empirical monaural loudness judgments serving as input. While NH showed binaural inhibition in line with the literature, binaural summation and its inter-subject variability were increased in HI, indicating the necessity for individualized binaural summation. Toward ILMs, a recent monaural loudness model was extended with the suggested binaural stage, and the number and type of additional parameters required to describe and to predict individual loudness were assessed. In addition to one parameter for the individual amount of binaural summation, a bandwidth-dependent monaural parameter was required to successfully account for individual spectral summation.

## Introduction

Being “too loud” is the most frequent descriptor for fitting problems with hearing aids (Jenstad et al., 2003), and current hearing aid fitting procedures take loudness into consideration (e.g., Moore and Glasberg, 1998; Byrne et al., 2001; Keidser et al., 2012). For instance, when deriving the widely used fitting formula NAL-NL1, loudness models were used to ensure that speech stimuli are not perceived louder by aided hearing impaired (HI) listeners than by normal hearing (NH) listeners (Byrne et al., 2001). Nevertheless, gains prescribed by NAL-NL1 or similar fitting procedures were still too high for many HI listeners (Keidser et al., 2012). This indicates that the loudness of HI listeners was underestimated by the loudness model and the prescribed gains were reduced in NAL-NL2 (Keidser et al., 2012).

Loudness perception differs significantly across individuals with similar audiometric hearing loss (Moore, 2000) and, to some extent, for NH listeners (e.g., Pieper et al., 2018). This suggests that loudness models with parameters based on averaged data, and with individualization of parameters for HI listeners inferred solely from their audiogram (e.g., Moore et al., 1999), might not be sufficient to predict individual loudness perception (Oetting et al., 2013; Pieper et al., 2018). Accordingly, if such models are involved in the first fitting of a hearing aid, subsequent manual adjustments are likely required. In order to improve individualized loudness predictions, existing parameters of loudness models need to be considered for individualization or additional parameter-controlled stages need to be introduced. Pieper et al. (2018) extended the physiologically motivated loudness model for average NH listeners for individualized loudness predictions of NH and HI listeners. In addition to typical assumptions like an expansive component (or reduced compression component) related to cochlear gain loss and an attenuation component (e.g., Launer, 1995; Derleth et al., 2001; Chalupper and Fastl, 2002; Moore and Glasberg, 2004; Chen et al., 2011a), they suggested a frequency-dependent post gain, potentially reflecting central gain mechanisms (for review, see Brotherton et al., 2015) to improve individual loudness predictions. Although the post gain improved the ability to fit the extended loudness model to individual loudness data for narrowband stimuli, predictions for broadband stimuli were not improved. Furthermore, their model was only applied to monaural stimuli. However, in realistic environments, sounds are typically perceived binaurally in addition to showing broadband properties, as observed for, e.g., speech and environmental noise.

With bilaterally aided HI listeners, it has been shown that binaurally presented broadband stimuli are perceived louder by aided HI listeners than by NH listeners at high levels, i.e., the uncomfortable level perceived as “too loud” is reached at lower levels (Oetting et al., 2018; van Beurden et al., 2020). For monaural presentation, this effect is smaller (Strelcyk et al., 2012; Oetting et al., 2016; Ewert and Oetting, 2018). Taken together, these findings suggest that binaural loudness summation can be affected by hearing loss and depend on the bandwidth of the stimulus. Parameters of binaural loudness summation might be related to physiological processes like the middle ear-muscle (MEM) reflex (Møller, 1962) or the medial olivocochlear (MOC) reflex (Berlin et al., 1993, 1995; Norman and Thornton, 1993; Guinan, 2006). Binaural loudness summation might as well be influenced by later stages of the central auditory system: Binaural inhibition was found in the inferior colliculus (see Li and Yue, 2002, for an overview) probably mediated in part by auditory neuronal stages prior to the inferior colliculus, such as the lateral superior olive (Finlayson and Caspary, 1991) or the dorsal nucleus of the lateral lemniscus (Faingold et al., 1993).

Most studies on binaural loudness summation use the loudness ratio between binaural loudness *N*_*B*_ and monaural loudness *N*_*M*_ in sones to quantify the amount of binaural loudness summation. The ratio *N*_*B*_/*N*_*M*_ had been assumed to be close to 2 based on the assumption that binaural loudness is the sum of the monaural loudness in sones (e.g., Hellman and Zwislocki, 1963; ANSI S3.4, 2007), while more recent studies have suggested *N*_*B*_/*N*_*M*_ < 2, i.e., binaural loudness in sones is less than twice the monaural loudness in sones (Zwicker and Zwicker, 1991; Sivonen and Ellermeier, 2006; Whilby et al., 2006; Epstein and Florentine, 2009). Current loudness models include a binaural inhibition stage to account for these findings (Moore et al., 2014, 2016): If assuming *N*_*B*_/*N*_*M*_ = 1.5, a wide variety of averaged NH loudness data can be successfully predicted (Moore et al., 2016). For HI listeners, binaural level differences for equal loudness (BLDELs) were (slightly) underestimated (Moore et al., 2014), indicating that the assumed ratio of *N*_*B*_/*N*_*M*_ = 1.5 might be too low to account for binaural loudness summation in HI individuals. Ewert and Oetting (2018) found higher ratios for HI listeners ($\frac{N_{B}}{N_{M}}=\;2.1\pm 0.5$, mean ± standard deviation) than for NH listeners ($\frac{N_{B}}{N_{M}}=\;1.7\pm 0.4$) using broadband stimuli. In combination, these results suggest that particularly in HI, individual differences in binaural loudness summation might exist.

The goal of this study is to develop a binaural loudness model that can be individualized for NH and HI listeners. Hearing aid fitting and development might benefit from individualized loudness models, enabling better adaptation of the processing to the individual needs, including model-based control of hearing aid signal processing to optimize loudness perception for arbitrary stimuli. Hereby, the critical question is how many and which parameters are required in addition to the commonly used cochlear gain or outer hair cell (OHC) loss and attenuation or inner hair cell (IHC) loss component, to allow for both the ability of the model to account for and to predict individual binaural loudness data in NH and HI listeners. Additional parameters should have a psychoacoustical or physiological motivation resulting in a structured functional model. In light of applicability for hearing aid development and fitting, loudness is modeled in four basic conditions covering the variety in bandwidth and binaurality occurring in natural sounds: (i) monaural narrowband, (ii) binaural narrowband, (iii) monaural broadband, and (iv) binaural broadband. For the model development and evaluation of this study, monaural and binaural loudness data were collected, focusing on narrowband stimuli with different center frequencies in order to be able to access the frequency dependency of binaural loudness summation. Additional loudness data were available from an earlier study of Oetting et al. (2016).

In a first experiment, a simplified binaural summation stage was devised and tested in a data-driven approach in which the binaural stage was applied directly to the measured monaural loudness data for the two ears of individual listeners. By this, binaural loudness summation can be assessed without relying on accurate loudness predictions for monaural stimulus presentation. The simplified binaural stage has a single parameter that controls the overall binaural gain. However, using monaural loudness data as input, this approach assumes that the binaural summation itself cannot be frequency- or bandwidth-dependent. Thus, in a second experiment, the monaural loudness model of Pieper et al. (2018) was extended with an augmented version of the above binaural summation stage where the binaural gain depends on the modeled internal excitation pattern after basilar membrane (BM) processing, which in turn depends on the bandwidth and level of the stimulus. Hereby, the modeled excitation pattern is influenced by individual properties of the peripheral auditory system, such as an individual OHC and IHC loss, and a central gain (Pieper et al., 2018). In order to improve monaural loudness predictions over those of Pieper et al. (2018), a bandwidth-dependent central gain is introduced into the monaural paths of the model prior to the binaural summation stage. Taken together, in addition to the frequency-dependent peripheral components OHC and IHC loss, commonly contained in HI loudness models, and the frequency-dependent post gain introduced in Pieper et al. (2018), four further frequency-independent parameters were introduced, which control a bandwidth-dependent monaural gain in each ear (two parameters), an overall binaural gain (one parameter), and the bandwidth dependency of the binaural gain (one parameter). The extended loudness model was then used to determine which of these individual parameters are required to describe loudness perception in the four basic conditions mentioned above. The improvement of the goodness of fit for each of the parameters was estimated in a hierarchic manner.

Suggestions are devised on which parameters and measurements are required to provide an individual loudness model applicable for hearing aid fitting and aided performance prediction.

## Model Extensions and Modifications

**Figure 1:**
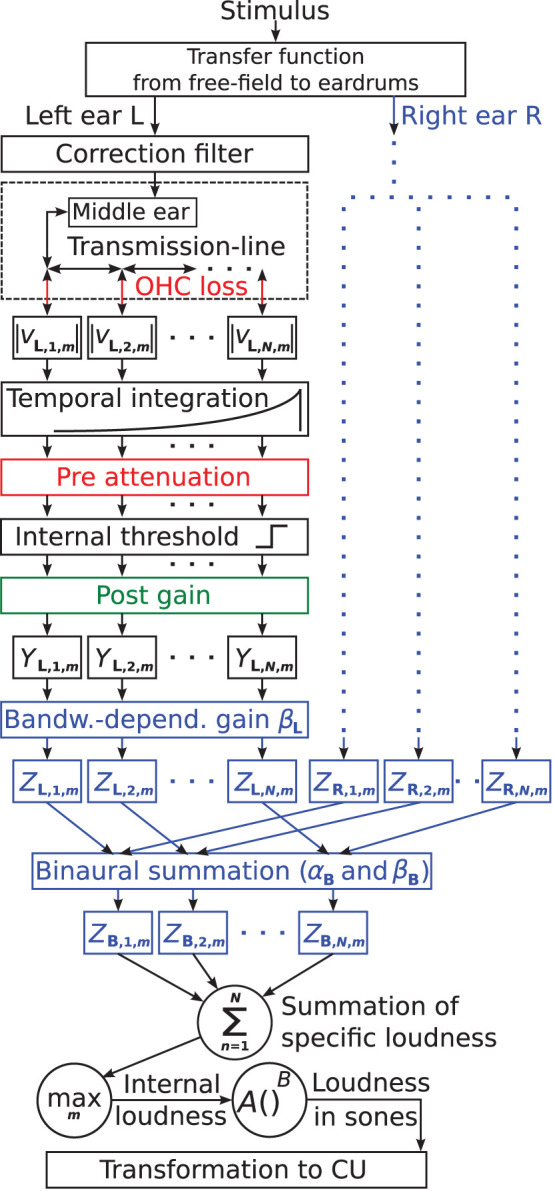
Block diagram of the individual loudness model based on Pieper et al. (2018). Model parts colored in red and green contain frequency-dependent parameters to account for individual hearing loss. Red indicates attenuation and green indicates amplification. Here, the suggested model extensions for further individualization are colored in blue: the parameters β_*L*_ and β_*R*_ control a monaural bandwidth-dependent central gain for the left and right ears, respectively. α_*B*_ controls the overall amount of binaural summation, and β_*B*_ controls the binaural summation depending on the bandwidth of the input signals *Z*_*L,n,m*_ + *Z*_*R,n,m*_.

The suggested binaural loudness model is based on the monaural loudness model of Pieper et al. (2018). Figure 1 shows the block diagram of the model. The colored parts contain model parameters used for individualization, with blue parts reflecting model extensions of this study. Here and in the following, subscripts L and R denote constants and variables of the left and right ear, respectively. Subscript B denotes constants and variables of the binaural summation stage. The model follows a signal processing chain structure where each stage receives input only from the previous stage.

The stimulus first passes through a fixed filter representing the transfer function from the sound source to the eardrums for free-field conditions. For frontal incident, the filter meets the ANSI S3.4 (2007) standard, in line with existing loudness models, e.g., Moore et al. (1997) and Chen et al. (2011b). If azimuth shifts from the frontal incidence were simulated, the same amplitude and phase shifts as for the stimuli were applied (see Section Apparatus, Procedure, and Stimuli).

The correction filter, attenuating low and amplifying high frequencies (see Pieper et al., 2016, 2018 for details), is applied to obtain a frequency-dependent absolute threshold according to ISO 389-7 (2005).

The middle ear transfer function is realized with a fixed finite impulse response filter that was fitted closely to the data of Puria (2003).

A physiologically plausible transmission-line model (TLM, e.g., Verhulst et al., 2018) of the cochlear simulates basilar membrane motion. The BM is divided in *N* = 1, 000 equidistant segments *n*. The cochlear gain of the BM can be reduced to account for OHC loss (indicated in red in Figure 1), typically referred to as compression loss component in the literature, accounting for steepening of the loudness function (loudness recruitment) as well as widening of auditory filter bandwidth. The TLM provides the segment velocities at the time steps *m* at a sampling frequency of 100 kHz. The absolute values (denoted |*v*_*L,n,m*_| for the left ear and |*v*_*R,n,m*_| for the right ear) are used as the input of the temporal integration stage.

Temporal integration is performed with a first-order low pass filter (time constant τ = 25 ms). Subsequently, the sampling frequency is reduced to 200 Hz.

IHC loss reflecting damage or loss of IHCs, often referred to as attenuation component in the literature, is implemented as linear attenuation prior to a constant internal threshold (referred to as pre attenuation, indicated red in Figure 1). The pre attenuation might be interpreted as a reduction of the summed spike rate of an adjacent IHC population, e.g., due to a reduction in the number of intact IHCs, attached synapses, or stereocilia (Pieper et al., 2018). The attenuation component shifts the entire loudness function to higher levels.

All signal parts below the internal threshold are set to 0, simulating the absolute hearing threshold. Thus, attenuation related to OHC and IHC loss prior to the internal threshold (shown in red) effectively increases the hearing threshold. The internal threshold might be interpreted as a specific summed spike rate, which has to be overcome in order to evoke responses in higher processing stages (Pieper et al., 2018).

The subsequent post gain (shown in green in Figure 1) is linear amplification applied to the signal part above the internal threshold, assumed to reflect effects of central gain (e.g., Heinz et al., 2005; Zeng, 2013). For HI listeners, a post gain exactly opposite to the pre attenuation counteracts the effect of the pre attenuation for high levels. This leads to the same uncomfortable level as in NH and steepening of the loudness function above the hearing threshold (see Figure 2 in Pieper et al., 2018). If the post gain is viewed as a part of the IHC loss component and depends on the pre attenuation, the current implementation of IHC loss as well as that of OHC loss are both functionally comparable to the respective components of other HI loudness models (e.g., Launer, 1995; Derleth et al., 2001; Chalupper and Fastl, 2002; Moore and Glasberg, 2004; Chen et al., 2011a). However, Pieper et al. (2018) demonstrated that the post gain is required to be a free parameter for both HI and NH listeners to account for individual differences in the steepness of loudness functions for narrowband stimuli.

**Figure 2:**
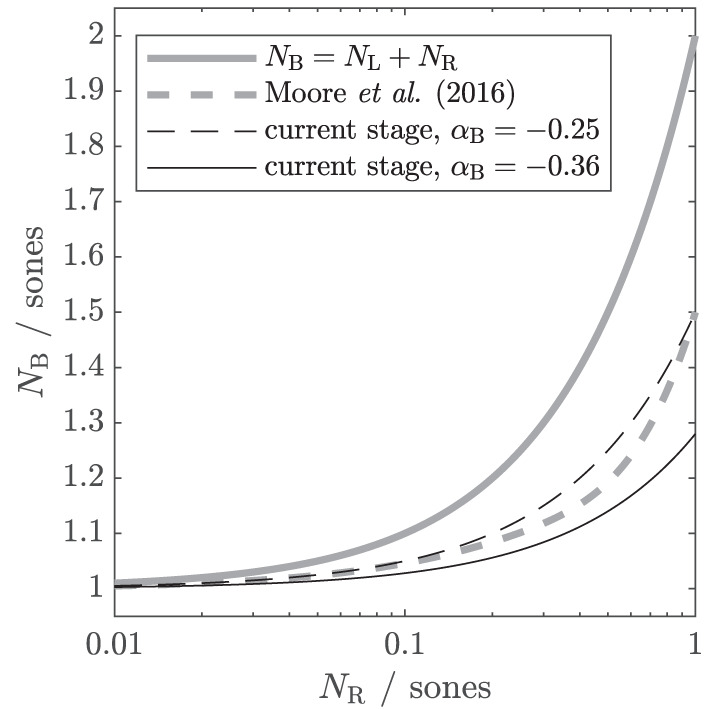
Different theoretical assumptions of binaural summation, depicted as input-output functions. The output is the binaural loudness in sones *N*_*B*_. The input is the monaural loudness for the right ear *N*_*R*_, while the monaural loudness for the left ear *N*_*L*_ is kept constant at 1 sone. In the suggested binaural stage, the individual amount of binaural summation can be controlled *via* a parameter α_*B*_. Gray solid line: the classical assumption that binaural loudness is the summed monaural loudness in sones. Gray dashed line: binaural stage of the loudness model of Moore et al. (2016). Black lines: simplified version (β_*B*_ = 0, *N*_*L*_ and *N*_*R*_ as input) of the current binaural stage with α_*B*_ = − 0.25 (dashed; comparable to Moore et al., 2016) and α_*B*_ = − 0.36 (solid; obtained from the first experiment of this study).

In Pieper et al. (2018), the output of the post gain stage for each BM segment is denoted as *Y*_*n,m*_ and is summed over the BM segments *n* at every time step *m* to yield the time-dependent internal loudness *I*_*m*_ for a single ear (summation of specific loudness). In the current binaural model, *Y*_*n,m*_ is calculated separately for each ear and is denoted as *Y*_*L,n,m*_ for the left ear and *Y*_*R,n,m*_ for the right ear.

At the output of the post gain stage, the model is extended (blue parts in Figure 1) by a monaural bandwidth-dependent central gain and a binaural summation stage. These extensions introduce four additional parameters that are considered for individualization:

- Two monaural parameters, β_*L*_, and β_*R*_, for the left and right ears to adjust the monaural bandwidth-dependent central gain individually (see Equation 1),
- One parameter that controls the overall amount of binaural inhibition α_*B*_, and
- One parameter that controls the bandwidth dependency of binaural inhibition β_*B*_.

In the following, equations that are applied in both ears separately are described only for the left side.

### Monaural Extension

In Pieper et al. (2018), the individually adjusted post gain did not improve the individual loudness predictions for broadband stimuli. No peripheral parameters (such as outer and middle ear transfer function, OHC and IHC loss, thresholds of BM compression) were identified, which quantitatively explain the remaining individual variations in spectral loudness summation. In principle, the physical BM properties could be individually altered in the TLM to change the auditory filter bandwidth and therefore affect the modeled spectral loudness summation. However, since auditory filters are already widened in HI models because of the OHC loss, and excitation patterns for narrowband stimuli already cover a large portion of the BM, further substantial changes in spectral loudness summation are not expected by such modifications (see, e.g., Zwicker and Scharf, 1965). Pieper et al. (2018) supposed that the medial olivocochlear reflex (Guinan, 2006) or more central mechanisms might be involved. These mechanisms might be altered as a consequence of hearing impairment and might therefore differ across ears. Therefore, as a first functional approach, an additional monaural bandwidth-dependent central gain [1 + β_*L*_ · *W*_*L,m*_ ] is introduced here (see Equation 1), which is multiplied with the output of the post gain stage *Y*_*L,n,m*_ in all segments of the BM at every time step *m* to obtain the final output of the monaural stage *Z*_*L,n,m*_ (see Figure 1). The gain can be individualized with the constant parameter β_*L*_. The bandwidth estimator *W*_*L,m*_ is calculated as a function of *Y*_*L,n,m*_ by dividing the average of *Y*_*L,n,m*_ across segments $({\bar{Y}}_{L,m}=\frac{1}{N}\sum_{n=1}^{N}Y_{L,n,m}$) by the mean of the absolute differences between *Y*_*L,n,m*_ and ${\bar{Y}}_{L,m}$ (Equation 2).

(1) $$\begin{array}{l} Z_{L,n,m}=\;\left[1+\beta_{L}\cdot W_{L,m}\;\right]\cdot Y_{L,n,m}, \end{array}$$

(2) $$\begin{array}{l} W_{L,m}=\frac{{\bar{Y}}_{L,m}}{\frac{1}{N}\sum_{n=1}^{N}|Y_{L,n,m}-{\bar{Y}}_{L,m}|}-\frac{1}{2}\cdot \frac{1}{1-\frac{1}{N}}. \end{array}$$

The second term $-\frac{1}{2}\cdot \frac{1}{1-\frac{1}{N}}\approx -\;0.5$ ensures that *W*_*L,m*_ = 0 at the hearing threshold for a narrowband signal, for which *Y*_*L,n,m*_ > 0 occurs at a single segment only. Above hearing threshold, the excitation pattern broadens with level, particularly for narrowband stimuli, resulting in bandwidth estimations *W*_*L,m*_ higher than 0. As a consequence, the bandwidth-dependent gain will alter the model output not only for broadband stimuli but for narrowband stimuli as well. However, as *W*_*L,m*_ grows exponentially with the width of *Y*_*L,n,m*_ (in contrast to the linear growth of other bandwidth estimators used in, e.g., Rennies et al., 2009; Oetting et al., 2018), good separation between narrowband and broadband signals is maintained despite the broadening of excitation pattern for narrowband stimuli. The same is independently introduced in the right ear, resulting in the two monaural parameters β_*L*_ and β_*R*_, respectively.

### Binaural Stage

The binaural stage sums *Z*_*L,n,m*_ and *Z*_*R,n,m*_ present at the monaural paths of each segment. The sum is multiplied with a binaural gain to obtain the output *Z*_*B,n,m*_:

(3) $$\begin{array}{l} Z_{B,n,m}=\left[1+\;\alpha_{B}V_{B,n,m}+\;\beta_{B}V_{B,n,m}W_{B,m}\right]\cdot \left[Z_{L,n,m}+Z_{R,n,m}\right], \end{array}$$

where *V*_*B,n,m*_ denotes the binaural difference of *Z*_*L,n,m*_ and *Z*_*R,n,m*_:

(4) $$\begin{array}{l} V_{B,n,m}=1-\frac{\left|Z_{L,n,m}-Z_{R,n,m}\right|}{Z_{L,n,m}+Z_{R,n,m}}. \end{array}$$

Equation 4 is a simplified version of the equation that is used in Oetting et al. (2018) to estimate the binaural loudness difference. Here, *V*_*B,n,m*_ equals 0 for monaural conditions (with either *Z*_*L,n,m*_ = 0 or *Z*_*R,n,m*_ = 0), in which case the stage does not alter loudness. *V*_*B,n,m*_ equals 1 if the signals *Z*_*L,n,m*_ and *Z*_*R,n,m*_ are identical in the monaural paths (diotic stimuli), and is between 0 and 1 if a signal is present in both monaural paths.

Two constant parameters α_*B*_ and β_*B*_ are used to individualize the binaural stage. α_*B*_ alters the gain as a function of the binaural difference *V*_*B,n,m*_. Binaural inhibition is modeled if α_*B*_ < 0, as the gain is lower (and smaller than 1) the higher *V*_*B,n,m*_ is, i.e., the more equal *Z*_*L,n,m*_ and *Z*_*R,n,m*_ are. β_*B*_ alters the gain as a function of the binaural difference and the binaural bandwidth estimator *W*_*B,m*_. For *W*_*B,m*_, the same bandwidth estimation as for the monaural stage (Equation 2) is applied where *Y*_*L,n,m*_ is replaced by the binaural sum *Z*_*L,n,m*_ + *Z*_*R,n,m*_. If β_*B*_ is set to 0 and if the signal is identical in the monaural paths (*V*_*B,n,m*_ = 1), α_*B*_ directly reflects the amount by which *Z*_*L,n,m*_ + *Z*_*R,n,m*_ is altered.

Finally, summation of specific loudness is performed to derive the time-dependent internal binaural loudness:

(5) $$\begin{array}{l} I_{m}=\frac{1}{N}\overset{N}{\underset{n=1}{\sum }}Z_{B,n,m} \end{array}$$

and transformed to loudness in sones by a power-law function and subsequently to loudness in categorical units CU by a non-linear transformation as described in the Appendix.

In order to test the binaural stage independently from the monaural stages of the loudness model, a slightly modified version of the suggested binaural stage was first used in a data-driven approach. This approach aims to predict binaural loudness from the monaural loudness measurements. For this, the empirically derived loudness in sones for monaural stimulus presentation *N*_*L*_ and *N*_*R*_ was used as input to the binaural stage, replacing the signals *Z*_*L,n,m*_ and *Z*_*R,n,m*_ of the monaural model paths. Given that only a single input value per ear exists and the bandwidth estimation *W*_*B,m*_ is unknown in this case, the binaural bandwidth-dependent gain in Equation 4 is deactivated by setting β_*B*_ to 0.

Figure 2 shows the input–output function of this simplified binaural stage in comparison to other assumptions for binaural loudness summation from the literature. The binaural loudness estimate *N*_*B*_ is shown as a function of *N*_*R*_ ranging from 0.01 to 1 sone, with *N*_*L*_ kept constant at 1 sone. If α_*B*_ is set to 0, the current binaural stage follows the classical assumption that the binaural loudness in sones *N*_*B*_ is simply the sum of the monaural loudness in sones (gray solid line, Hellman and Zwislocki, 1963; ANSI S3.4, 2007). If α_*B*_ is set to −0.25 (black dashed line), the input–output function is comparable to that of the binaural stage of Moore et al. (2016, gray dashed line), which accounts for a wide variety of averaged NH loudness data. If *N*_*R*_ is much lower than *N*_*L*_ (e.g., *N*_*R*_ = 0.1, *N*_*L*_ = 1), the contribution of *N*_*R*_ to *N*_*B*_ is still further reduced by the binaural inhibition, resulting in *N*_*B*_ = 1.05, i.e., for large loudness differences between ears, the softer ear hardly contributes to binaural loudness.

## Methods

### Listeners

Eight NH listeners and eight HI listeners with slight-to-moderate sensorineural hearing loss (SNHL) participated in the study. The NH listeners had audiometric thresholds of 15 dB HL or better at the test frequencies 0.25, 0.5, 1, 2, 4, and 6 kHz. The mean audiometric thresholds and standard deviation of the HI group were 23 ± 11, 33 ± 12, 40 ± 11, 48 ± 18, 61 ± 12, and 59 ± 17 dB HL at the six test frequencies, respectively.

### Apparatus, Procedure, and Stimuli

Adaptive categorical loudness scaling (Brand and Hohmann, 2002) was performed to obtain loudness estimates for narrowband low-noise noise (LNN) stimuli with center frequencies of 0.25, 0.5, 1, 2, 4, and 6 kHz and a bandwidth of one-third octave. In comparison to other loudness measurement procedures, loudness scaling offers an easy and fast method applicable in a clinical context. The listener judges loudness on a scale with 11 labeled and unlabeled categories. Labeled categories are “no heard” (0 CU), “very soft” (5 CU), “soft” (15 CU), “medium” (25 CU), “loud” (35 CU), “very loud” (45 CU), and “too loud” (50 CU). In between the categories “very soft” and “very loud,” the categories alternate between labeled and unlabeled categories (10, 20, 30, and 40 CU). In addition to the widely used narrowband stimuli, broadband stimuli were presented to a subset of four NH and four HI listeners. The broadband stimuli, referred to as international female (IF) speech noise, were stationary speech-shaped noise generated from the international speech test signal (Holube et al., 2010). The spectral shape of the signal is the same as the (international) long-term average speech spectrum for females (Byrne et al., 1994). IF noise stimuli were presented unaided or aided. For the aided condition, the monaural narrowband loudness compensation, as described in Oetting et al. (2016), was employed: The spectrum of the stimulus is divided in adjacent frequency bands, and the monaural loudness of the HI listener is restored to average NH loudness in each band. For HI listeners, this resulted in higher amplifications of frequency bands with lower power, resembling the non-linear, level-dependent gain in a hearing aid. Exactly the same procedure was applied for the individual NH listeners (with considerably smaller adjustments than required for HI) and is also referred to as “aided” for NH.

For each listener, the stimuli were presented monaurally in the left, in the right ear, and diotically *via* headphones (Sennheiser HDA200). The listeners were seated in a sound attenuating booth, and responses were collected from a touchscreen connected to a personal computer. Signal generation and experimental control were performed in MATLAB using the AFC package (Ewert, 2013). The headphones were free-field equalized. For the diotic presentation, this means a simulated frontal incident of the sound waves. For the IF noise stimuli, additional azimuth shifts of the incident to the left by 60° and to the right by 60° were simulated. For this, frequency-dependent interaural level and phase differences were applied derived from the interaural differences in the head-related binaural impulse responses for ±60° re frontal incidence of the database from Kayser et al. (2009).

Headphone calibration and equalization ensured level differences between the left and right ears in the binaural conditions of <3 dB for all tested frequencies. In the following, we refer to the outcome of the respective measurements performed in this study as Dataset 1.

As a second set of data (referred to as Dataset 2), categorical loudness scaling data of eight NH and 10 HI listeners of Oetting et al. (2016) were used in this study^1^. The monaural data for the left ear were the same as those used in Pieper et al. (2018). Data for the same narrowband LNN stimuli as used in this study were available but only for monaural presentation. However, data for a narrowband uniform exciting noise (UEN1, Fastl and Zwicker, 2007) with a center frequency of 1,370 Hz and a bandwidth of 210 Hz were available for both monaural aided and binaural aided conditions. For narrowband stimuli, aided conditions imply that not the same level but the same (monaural) loudness was presented to each ear. Broadband stimuli were the same IF noise stimuli used in this study. For NH listeners, only data for unaided conditions are available.

### Estimation of Loudness Functions

The fitting procedure “BTUX,” as recommended by Oetting et al. (2014), was performed to derive individual loudness functions as well as the hearing thresholds from the raw data of Datasets 1 and 2. BTUX fits a loudness function proposed by Brand and Hohmann (2002) that consists of two straight lines connected with a Bezier curve to the loudness data. First, the hearing threshold level is estimated from all raw data points and assumed to correspond to loudness of 2.5 CU between categories 0 CU (“not heard”) and 5 CU (“very soft”). The function is then fitted to all data points above threshold with the prescribed threshold level at 2.5 CU using the least-squares method in the direction of the level (X-direction). If <5 data points are available between 35 and 50 CU, the slope of the upper straight line is set to a fixed value. The hearing threshold estimations of BTUX were used in the following experiment II.

The reference NH functions used for the narrowband loudness compensation (aided conditions) were the same as those shown in **Table 2** in Oetting et al. (2016). These functions are the average loudness functions across nine NH listeners.

### Experiment I: Binaural Loudness Summation and Data-Driven Binaural Stage

In the first experiment, the binaural summation ratios *R* were calculated for the individual loudness functions from Datasets 1 and 2. *R* was defined as:

(6) $$\begin{array}{l} R=\frac{2N_{B}}{N_{L}+N_{R}}, \end{array}$$

where *N*_*B*_ denotes the binaural loudness in sones and *N*_*L*_ and *N*_*R*_ the monaural loudness for the left and right ear, respectively. *R*>2 indicates that the binaural loudness *N*_*B*_ is higher than the sum of the monaural loudness values. This is referred to as binaural excitation in the following. *R* < 2 indicates binaural inhibition (e.g., Moore et al., 2016). Given that loudness had been measured in CU, the CU values were transformed to sone values with the five-parameter cubic function as suggested by (Heeren et al., 2013). Contrary to the procedure commonly used in the literature, where loudness categories are calculated for fixed sound pressure levels, loudness ratios were calculated for the given loudness categories of the binaural (diotic) condition. This allows the comparison of loudness ratios across listeners for, e.g., medium loudness (25 CU) or the “very loud” category (45 CU) close to the uncomfortable level. To obtain the ratio *R*, the level at which the individual binaural loudness function yields the desired CU value was determined. For that level, the respective CU values of the individual monaural loudness functions were obtained. These binaural and monaural CU values at an equal level were then transformed to sone values.

The value of *R* is comparable to the binaural summation ratios given in earlier studies if the same loudness was present in both ears, i.e., *N*_*L*_ equals *N*_*R*_. For the diotic data of this study, loudness can be unequal in both ears, in particular in the case of unaided asymmetric hearing loss, so that the individual values of *R* are expected to be closer to 2 compared with those found in the literature. Thus, the ratio *R* (Equation 6) does not directly indicate binaural inhibition or excitation if unequal loudness occurs across the ears. In order to enable comparisons with the literature and across listeners and conditions in the case of unequal loudness across ears, the simplified binaural summation stage (operating on the monaural empirical loudness data in sones, see model extensions above) can be used to derive “corrected” ratios as would have been observed for equal loudness in both ears (see below).

In order to quantify the possible benefit of individualizing the binaural stage, the simplified binaural summation stage (which was fitted to the individual binaural summation ratios for each listener) was compared with a binaural stage that only considers the average (non-individualized) binaural gain derived for NH. After transformation of the stage output in sones back to CUs, the error between CUs inferred with the individualized and non-individualized stage and measured loudness functions was calculated and compared.

The individualized implementation of the binaural stage was realized by allowing for individual values of the binaural gain α_*B*_. Individual values were derived by fitting the loudness ratios calculated from the model outputs ${\hat{R}}_{s,c}$ to the empirically derived loudness ratios *R*_*s,c*_ for the LNN stimuli, using α_*B*_ as the fit parameter. The error function that was minimized in the fit was:

(7) $$\begin{array}{l} \overset{6}{\underset{s=1}{\sum }}\overset{50}{\underset{c=15}{\sum }}{\left(\mathrm{lg}\left({\hat{R}}_{s,c}\right)-\mathrm{lg}\left(R_{s,c}\right)\right)}^{2}, \end{array}$$

where *s* = 1, 2, …, 6 is the number of the six LNN stimuli, and *c* = 15, 20, …, 50 is the loudness category of the empirical loudness function for diotic conditions before transformation to loudness in sones. 5 and 10 CU were excluded in experiment I to ensure that the monaural loudness was always above the hearing threshold. The non-individualized stage used the mean value across the individual values for α_*B*_ of the NH listeners.

The amount of binaural inhibition can be assessed more directly if the summation ratios are derived for equal monaural loudness in both ears. Since the hearing thresholds of the HI listeners are usually less symmetric than for the NH listeners, unequal loudness in both ears is to be expected in particular for unaided HI listeners if measurement conditions are diotic. Unequal loudness generally reduces the effect of binaural inhibition or excitation, leading to ratios of *R* closer to 2 compared with the case where loudness is equal in both ears^2^. Thus, in addition to the calculations of *R* for a diotic condition as given above (Equation 6), binaural summation ratios were estimated for equal loudness in both ears: substitution of the binaural loudness *N*_*B*_ in Equation 6 with the simplified version of Equation 3 (internal loudness *Z*_*n,m*_ replaced by loudness in sones *N*, β_*B*_ = 0, see model extensions above) and inserting equal loudness *N*_*L*_ = *N*_*R*_ yields the “corrected” binaural summation ratio for assumed equal loudness in both ears:

(8) $$\begin{array}{l} R=2\cdot \left(1+\alpha_{B}\right). \end{array}$$

To obtain the binaural summation ratio for assuming equal loudness for a certain stimulus, the fit procedure described above was applied to the stimulus in isolation to determine α_*B*_ in Equation 8. It should be noted that the binaural summation ratios resulting from this procedure are assumed to be independent of the loudness category.

In the fits, α_*B*_ had a lower limit of α_*B*_ = − 0.5. This constraint ensured that binaural loudness is not lower than monaural loudness in the stage predictions as well as in the ratios for equal loudness (*R* ≥ 1 in Equation 8).

### Experiment II: Individual Parameters to Describe Monaural and Binaural Loudness

In the second experiment, the complete loudness model was individualized for each listener. In contrast to the isolated simplified binaural stage in experiment I, the loudness model accounts for the auditory preprocessing of the stimuli before they enter the binaural stage. Auditory preprocessing includes the frequency-place transformation on the BM, and thus spectral and bandwidth properties of the stimuli are available to the binaural summation stage and their effect on binaural loudness summation can be assessed.

Similar to experiment I, different model versions were tested for their ability to account for the empirical loudness data. Each version added an additional free parameter. In order to determine the individual parameter values, the individualized models were fitted to measured data for appropriate selected measurement conditions, e.g., monaural broadband data were added to the selection once the bandwidth-dependent individual monaural gain was enabled. The remaining loudness data were then predicted with the individualized models. The non-linear correlation coefficient (ncc), the root mean squared error (rmse), and the bias (bias) were used as performance measures. These measures are based on the level differences between modeled and measured loudness functions at a certain CU. The non-linear correlation coefficient ncc was calculated as:

(9) $$\begin{array}{l} ncc=1-\frac{\sum_{s}\sum_{c=5}^{50}{\left(L_{s,c}-{\hat{L}}_{s,c}\right)}^{2}}{\sum_{s}\sum_{c=5}^{50}{\left(L_{s,c}-\bar{L}\right)}^{2}}. \end{array}$$

$\bar{L}$ denotes the mean of the empirically derived levels *L*_*s, c*_ across all stimuli *s* and 10 categories *c* = 5, 10, …, 50. ${\hat{L}}_{s,c}$ are the respective model predictions. The category 0 CU was excluded as the model output is 0 CU for all levels below the hearing threshold. If all predicted levels match the empirically derived levels, i.e., ${\hat{L}}_{s,c}$ equals *L*_*s,c*_ for all *s* and *c*, *ncc* = 1 is obtained. If ncc equals 0, the predictions are as good as with $\bar{L}$ as predictor. Additionally, the adjusted ncc' was calculated when using all stimuli *s* to account for the number of individualized parameters *p*, i.e., the degrees of freedom of the model:

(10) $$\begin{array}{l} ncc^{\prime }=1-\left(1-ncc\right)\frac{n-1}{n-p-1}, \end{array}$$

where *n* = 10 · *s* is the number of observations. The adjusted ncc' was not calculated for a single stimulus where the number of parameters is higher than the number of observations.

The root mean square error rmse estimates the average deviation in dB between model and data:

(11) $$\begin{array}{l} rmse=\sqrt{\frac{1}{10}\overset{50}{\underset{c=5}{\sum }}{\left(L_{c}-{\hat{L}}_{c}\right)}^{2}}. \end{array}$$

The bias was calculated to identify systematic offsets in dB:

(12) $$\begin{array}{l} bias\;=\frac{1}{10}\overset{50}{\underset{c=5}{\sum }}\left(L_{c}-{\hat{L}}_{c}\right). \end{array}$$

Positive bias values indicate that the predicted loudness function is on average shifted to higher levels compared with the empirically derived loudness function (loudness is on average underestimated). rmse and bias were calculated for each stimulus in isolation.

The extension of the model with a binaural summation stage made it necessary to refine fixed parameters of the final transformations to loudness in sones and CU in the model (see Appendix). The procedure performed to refine those parameters also resulted in a non-individualized binaural stage modeling the average NH inhibition of the NH listeners in Datasets 1 and 2.

The following four model versions were considered, which incorporate a successively increasing number of free parameters in the above-described monaural and binaural stages:

1. Binaural stage with average NH binaural inhibition:
  - Model version 1 is the loudness model of Pieper et al. (2018), modified to account for average NH binaural inhibition. As in Pieper et al. (2018), the individual OHC and IHC losses were derived from the hearing threshold, and the lower slope of the loudness functions for monaurally presented narrowband LNN stimuli at frequencies 0.25, 0.5, 1, 2, 4, and 6 kHz. The cochlear gains of the TLM were set to account for OHC loss, and the pre attenuations were set to account for the IHC loss. The monaural post gains were fitted to the loudness functions for the monaural LNN stimuli. These individualization steps were performed for each ear separately. No monaural or binaural bandwidth dependencies were assumed, i.e., β_*L*_ = β_*R*_ = β_*B*_ = 0. The above-mentioned non-individualized binaural stage was used to account for average NH inhibition.
2. Addition of individualized bandwidth-dependent gain in the monaural paths:
  - The bandwidth-dependent gain in the monaural paths was individualized by adding β_*L*_ and β_*R*_ to the set of free parameters and by adding the monaural loudness data for the aided IF noise stimuli with frontal incidence to the targeted empirical data^3^. For the NH data of Dataset 2, no aided conditions were available; thus, the unaided IF noise stimuli were used. As new data were added to the fit, the weightings in the error function needed to be reconsidered^4^. The same binaural stage as in model version 1 was used.
3. Individualized gain of the binaural stage, independent of bandwidth:
  - Similar to experiment I, α_*B*_ was used as the free parameter to fit the model to the diotic/binaural narrowband loudness functions. For the listeners measured in this study, the data from Dataset 1 were used for the six diotic LNN stimuli. From Dataset 2, only one binaural narrowband stimulus was available per group: aided UEN1 for HI and unaided UEN1 for NH.
4. Individualized bandwidth-dependent gain of the binaural stage:
  - Here β_*B*_ was considered as a free parameter in addition to α_*B*_ in the binaural stage, and the loudness function for the aided binaural IF noise stimulus was added to the targeted empirical data. In order to ensure equal weighting of the narrowband and broadband data, the error for the IF noise stimulus was weighted by the number of narrowband stimuli used, which is 6 for Dataset 1 and 1 for Dataset 2. Again, for the NH listeners of Dataset 2, only unaided conditions were available and used.

It should be noted that adjustments of model parameters to the data are referred to as “fitting.” The modeled loudness data are only referred to as model “prediction” if the empirical data for the same stimulus were not used in the process of fitting model parameters.

## Results

### Experiment I: Binaural Loudness Summation and Data-Driven Binaural Stage

**Figure 3:**
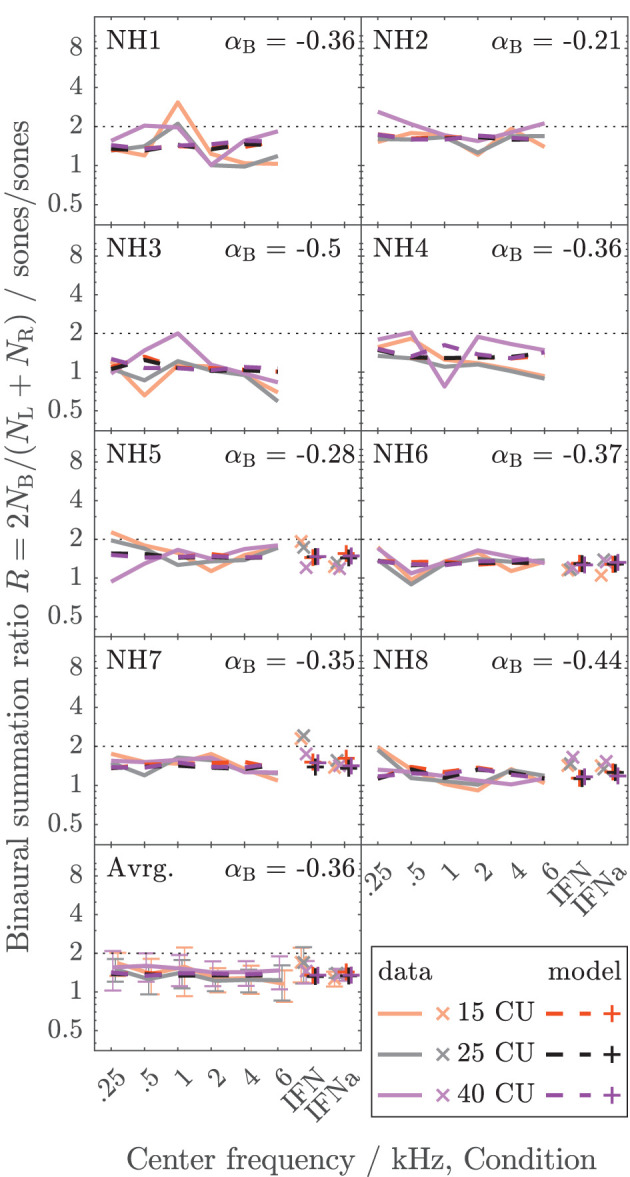
Loudness ratios *R* = 2*N*_*B*_/(*N*_*L*_ + *N*_*R*_) between loudness for diotic presentation *N*_*B*_ in sones and summed monaural loudness *N*_*L*_ + *N*_*R*_ in sones for the NH listeners of Dataset 1. The lines indicate diotically presented narrowband LNN stimuli with different center frequencies (0.25, 0.5, 1, 2, 4, and 6 kHz). Broadband IF noise stimuli with frontal incidence for unaided (IFN) and aided (IFNa) conditions are indicated with symbols. The colors indicate the loudness in CU for the diotic condition (orange: 15 CU, “soft,” gray/black: 25 CU, “medium,” purple: 40 CU, “loud–very loud”). The empirical data are shown as solid lines (narrowband stimuli) and x symbols (broadband stimuli) in lightened colors. Dashed lines and + symbols indicate the respective model calculations of the individualized binaural stage (the individual values for α_*B*_ are given for each listener). The bottom panel shows the averaged data across the NH listeners. Standard deviations are indicated with error bars.

**Figure 4:**
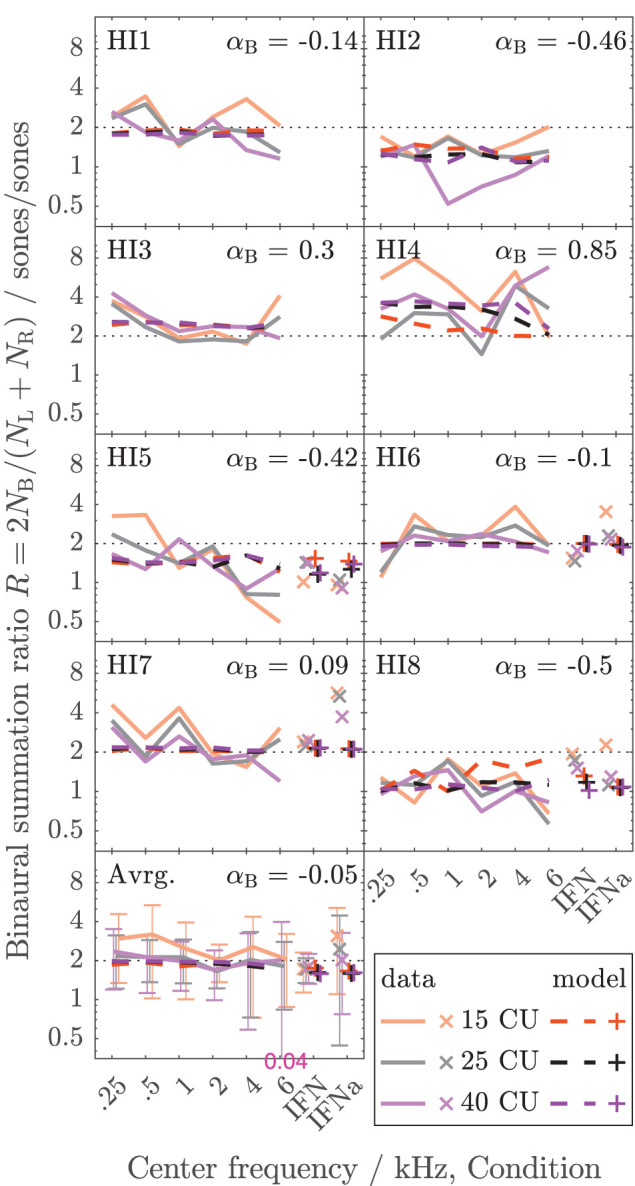
As Figure 3 but for the HI listeners of Dataset 1.

The data collected here characterize binaural loudness summation for narrowband stimuli with relatively fine frequency resolution for a wide range of frequencies (0.25, 0.5, 1, 2, 4, and 6 kHz) and for the whole level range from hearing threshold to or close to an uncomfortable level, as covered by the loudness functions. In addition, binaural loudness summation for broadband stimuli (IF noise aided and unaided) was assessed. The raw loudness data and the loudness functions are provided in the Supplementary Material. Here, Figures 3, 4 show the inferred loudness ratios *R* in sones/sones from the data for the NH and HI listeners, respectively. The empirically derived ratios of the NH listeners are usually lower than 2, indicating binaural inhibition in all the NH listeners (solid lines and x symbols of lightened colors). The values are similar across the NH listeners with the exception of NH3, whose data show basically no binaural summation (*R* close to 1). Nevertheless, as for the other NH listeners, the ratios for NH3 show no dependency of *R* on the loudness region (“soft,” “medium,” “loud–very loud” at 15, 25, and 40 CU, respectively), indicated by the loudness of the diotically presented stimulus (orange: 15 CU, gray: 25 CU, purple: 40 CU), and therefore *R* also does not depend on the stimulus level, as previously found by Marozeau et al. (2006). In some of the NH listeners, some unsystematic variation of the ratios with the stimulus frequency is observed. In contrast to the NH listeners in Figure 3, the ratios for the HI listeners in Figure 4 vary considerably across listeners and within listeners across stimuli. The ratios for HI3, HI4, and HI7 are predominantly higher than 2, indicating binaural excitation. Occasionally, quite high ratios are observed for few frequencies and mostly low levels (and low loudness categories) close to the hearing threshold (maximum ratio *R* = 7.9 at 0.5 kHz, 15 CU for HI4). On average, across the listeners (bottom panels), the ratios of NH and HI decrease slightly with increasing frequency.

For the NH listeners (Figure 3), the binaural model stage (dashed lines and + symbols) can be closely fitted to the ratios across all frequencies and loudness regions *via* parameter α_*B*_. For the HI listeners (Figure 4), the fit of the binaural model stage (dashed lines and + symbols) shows deviations to the empirically derived loudness ratios (solid lines and x symbols), particularly in the low loudness region (orange lines and symbols). However, because low loudness categories cover a high loudness range in sones if sones are plotted on a logarithmic scale (see Figure 3 in Heeren et al., 2013), ratios inferred after transformation to loudness in sones at low loudness categories are most sensitive to inconsistencies in the response of the listener and any biases in the method^5^. This is particularly true for the steep loudness functions found in HI listeners at low loudness categories (see, e.g., Oetting et al., 2016). Conversely, in the low loudness region, high deviations of the loudness ratios translate into relatively low deviations for the modeled binaural loudness in CUs.

**Figure 5:**
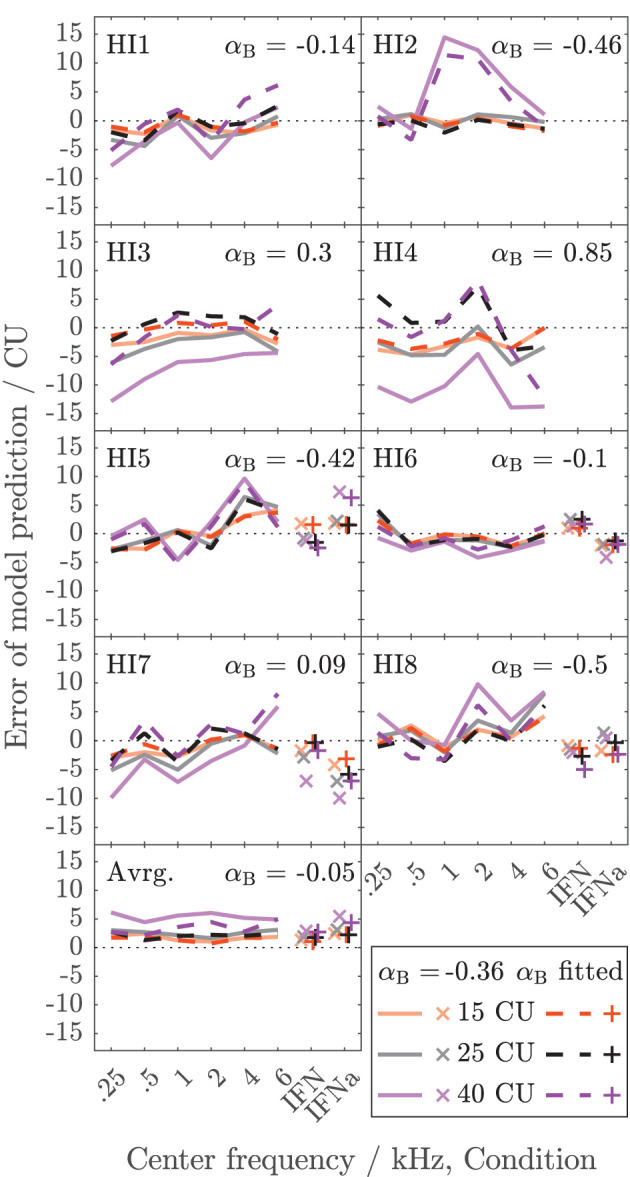
Deviations between model calculations of binaural loudness in CUs and empirical binaural loudness data in CUs for the HI listeners of Dataset 1. As in Figure 4, the color indicates the loudness of the diotic signal. Dashed lines and x symbols are for the individualized binaural model stage (see Figure 4). Solid lines and + symbols of lightened color are for the model using average NH binaural inhibition (Figure 3, mean of α_*B*_ = −0.36). The bottom panel shows the averaged *absolute* errors across listeners.

Figure 5 shows the error of the binaural summation stage if the modeled binaural loudness in sones is transformed back to CUs (HI listeners only). Dashed lines and + symbols indicate the errors for the fits of the binaural stage *via* α_*B*_, i.e., the individualized binaural stage for which the modeled loudness ratios are shown as dashed lines in Figure 4. Solid lines and x symbols indicate errors if α_*B*_ was fixed to the average value α_*B*_ = − 0.36 of the NH listeners, i.e., for the non-individualized binaural stage using average NH binaural inhibition. Errors in CUs are indeed low for the low loudness region. The absolute errors are lower than 5 CU for (binaural) loudness values of 15 CU (orange lines and symbols). The errors increase with loudness (gray: 25 CU, purple: 40 CU), as reflected by the mean values of the absolute errors across listeners shown in the bottom panel of Figure 5.

Individualized binaural summation reduces the errors of the modeled loudness for individual HI listeners at the high loudness region. This is shown by a comparison of the errors at 40 CU for the individualized binaural stage (purple dashed lines and + symbols) and the errors for the average NH binaural inhibition (purple solid lines and × symbols): For HI3 and HI4, the individualization reduces these errors by approximately 10 CU or two loudness categories. For HI7, these errors are reduced by more than a loudness category (5 CU) for the narrowband LNN stimuli with low center frequencies and for the broadband IFN stimuli. From the subgroup for which broadband data are available (HI 5–8), the two listeners HI6 and HI7 show decreased binaural inhibition for the narrowband stimuli (i.e., the fit to the narrowband data resulted in parameters α_*B*_ = −0.1 and α_*B*_ = 0.09, respectively). For these listeners, the accuracy of the binaural broadband predictions is increased (+ symbols in Figure 5) compared with no individualization (α_*B*_ = −0.36, × symbols at “IFNa” label in Figure 5). However, ratios inferred with the binaural model stage are similar for all stimuli within a listener. Thus, the stage does not account for, e.g., the frequency dependencies of binaural summation ratios in HI2 (compare purple solid with a dashed line in Figure 4 and see purple dashed line in Figure 5) or the increased binaural summation for the aided broadband stimulus (IFNa) in HI7 (compare x with + symbols at “IFNa” label in Figure 4).

The model fits are mostly determined by high loudness categories and almost not affected by low loudness categories, as indicated by the differences in error between fitted (dashed lines and + symbols) and unfitted (solid lines and x symbols) models in Figure 5. These differences are highest for high loudness categories (purple) and almost nonexistent for low loudness categories (orange). This is beneficial for the ratios for “assumed equal loudness between ears” addressed below, which are based on the model fits, because, as mentioned above, the ratios inferred for low loudness categories are most sensitive to inconsistencies in the response of the listener and any biases in the method.

Tables 1, 2 show the binaural summation ratios for each stimulus in isolation averaged across categories (15 to 50 CU) and NH or HI listeners. Table 1 shows the resulting ratios (mean ± standard deviation across listeners) for the data collected in this study and discussed above (Dataset 1) and Table 2 for the additional Dataset 2 provided by Oetting et al. (2016). In both tables, columns 4 and 5 list the ratios averaged across NH and HI listeners, respectively. As in Figures 3, 4, the ratios listed in the upper half of the table are for diotic conditions.

**Table 1:** Binaural summation ratios *R* averaged (mean ± standard deviation) across listeners of Dataset 1 (column 4: NH, column 5: HI) and across categories (15–50 CU).

**Stimulus type**	**Freq./kHz**	**Aided**	**Binaural summ. ratio** ***R***		**Variance *p***	**Mean *p***
			**NH**	**HI**		
**ORIGINAL DIOTIC MEASUREMENT CONDITION**						
Narrowband LNN (eight listeners)	0.25	No	1.56 ± 0.30	2.40 ± 1.09	**0.002**	0.071
	0.5	No	1.43 ± 0.29	2.29 ± 1.07	**0.045**	0.061
	1	No	1.47 ± 0.31	2.10 ± 0.82	0.054	0.073
	2	No	1.30 ± 0.21	1.73 ± 0.53	**0.030**	0.061
	4	No	1.32 ± 0.20	2.07 ± 1.38	**0.046**	0.175
	6	No	1.31 ± 0.30	2.01 ± 1.43	0.072	0.217
	ANOVA	No	1.40 ± 0.28	2.10 ± 1.06	**0.008**	0.054
Broadband IFN (four listeners)		No	1.54 ± 0.33	1.73 ± 0.44	0.605	0.529
		Yes	1.34 ± 0.10	2.33 ± 1.57	0.056	0.298
	ANOVA		1.44 ± 0.25	2.03 ± 1.12	0.092	0.294
**ASSUMED EQUAL LOUDNESS IN BOTH EARS**						
Narrowband LNN (eight listeners)	0.25	No	1.46 ± 0.32	2.35 ± 1.20	**0.001**	0.078
	0.5	No	1.35 ± 0.30	2.24 ± 1.13	**0.023**	0.063
	1	No	1.39 ± 0.30	1.97 ± 0.95	**0.024**	0.140
	2	No	1.21 ± 0.19	1.76 ± 0.63	**0.018**	**0.045**
	4	No	1.22 ± 0.23	2.01 ± 1.55	0.069	0.196
	6	No	1.22 ± 0.27	2.65 ± 3.80	0.055	0.326
	ANOVA	No	1.31 ± 0.27	2.16 ± 1.79	**0.008**	0.087
Broadband IFN (four listeners)		No	1.49 ± 0.32	1.55 ± 0.63	0.322	0.883
		Yes	1.27 ± 0.09	2.69 ± 2.32	**0.045**	0.308
	ANOVA		1.38 ± 0.25	2.12 ± 1.69	0.050	0.347

*The upper half of the table shows the results for the diotic measurement condition. The lower half shows the ratios for assumed equal loudness in both ears (see Method section of experiment I). The first column shows the stimulus type. Stimuli were unaided narrowband LNN with center frequencies given in column 2 (eight listeners) or broadband IFN for unaided and aided conditions (four listeners). Column 6 shows the p-value of Levene's test for equal variances between groups. Column 7 shows the p-value for equal means of Welch's t-test. The rows labeled ANOVA in column 2 show the respective mean values for the NH and HI groups (columns 3 and 4). Here, the p-values for the main effect of listener group (NH or HI) for the absolute deviation statistic of the ratios according to Levene and for the ratios are provided in columns 6 and 7. Significant differences (p < 0.05) are shown in bold*.

**Table 2:** Binaural summation ratios averaged across listeners of Dataset 2.

**Stimulus type**	**Freq./kHz**	**Aided**	**Binaural summ. ratio** ***R***		**Variance *p***	**Mean *p***
			**NH (eight listeners)**	**HI (10 listeners)**		
**ORIGINAL DIOTIC MEASUREMENT CONDITION**						
Narrowband UEN1	1.37	No	1.38 ± 0.16			
		Yes		1.72 ± 0.56		
Broadband IFN		No	1.93 ± 0.35	2.86 ± 1.01	**0.022**	**0.020**
		Yes		3.37 ± 2.06		
**ASSUMED EQUAL LOUDNESS IN BOTH EARS**						
Narrowband UEN1	1.37	No	1.28 ± 0.16			
		Yes		1.68 ± 0.69		
Broadband IFN		No	1.79 ± 0.35	2.97 ± 1.27	**0.014**	**0.018**
		Yes		3.65 ± 2.45		

*Narrowband stimuli were UEN1 with a center frequency of 1.37 kHz and were presented unaided for the NH listeners (column 4) but aided for the HI listeners (column 5). As both measurement conditions should have led to near equal loudness at the ears (see Method section of experiment I), the values for assumed equal loudness in the ears (lower half of table) are similar to the original values (upper half of table). However, this does not hold for the broadband IFN stimuli. The broadband conditions are the same as those in Table 1, but the results are based on a bigger listener pool (eight NH listeners and 10 HI listeners instead of four NH and four HI listeners). Column 6 shows the p-value of Levene's test for equal variances between groups. Column 7 shows the p-value of Welch's t-test. Significant differences (p < 0.05) are shown in bold*.

In both datasets, the standard deviations across the HI listeners (column 5) are more than twice as high as across the NH listeners (column 4) for all diotic conditions, indicating a higher inter-subject variability of binaural summation for the HI listeners. For the narrowband stimuli in Dataset 1, this observation is confirmed by a significant main effect of listeners group (*p* < 0.05) performing a two-way mixed-design ANOVA applied to Levene's absolute deviation estimate for each condition (column 6 of the upper part of Table 1 in the row labeled with ANOVA). For the individual narrowband LNN stimuli, Levene's test for equal variances between groups (NH and HI listeners) showed a significant difference in variances (*p* < 0.05) for most of the center frequencies (0.25, 0.5, 2, and 4 kHz) as well as for the broadband unaided IFN stimulus in Dataset 2 (column 6 in Table 2). Here and in the following, no correction for multiple comparisons was applied, as they were performed following a significant main effect of the ANOVA omnibus test.

In both datasets, the average binaural summation ratios are higher for the HI listeners than for the NH listeners, again for all diotic conditions: However, a significant main effect of the listeners group (*p* < 0.05) was not found performing a two-way mixed-design ANOVA (column seven in the row labeled with ANOVA). Significant differences in the average ratios (*p* < 0.05) between the groups were found for unaided IFN in Dataset 2 (Welch's *t*-test, column seven).

The lower halves of both Tables 1, 2 list the ratios for assumed equal loudness in both ears as described in the Method section of experiment I. The ratios for assumed equal loudness in the ears averaged across the NH listeners of Dataset 1 differ by 5.6, 5.8, 5.3, 5.2, 6.7, and 10.7% (median across absolute percentages) from the ratios for the original diotic measurement conditions at the stimuli center frequencies of 0.25, 0.5, 1, 2, 4, and 6 kHz, respectively. For the HI listeners of Dataset 1, the respective values are 5.7, 5.2, 7.1, 7.8, 9.2, and 15.3 %, and therefore similar to the values of the NH listeners at low frequencies but increased for medium to high frequencies.

For the NH listeners, inter-subject variability of the ratios for assumed equal loudness in ears is similar to the ratios for diotic conditions (compare standard deviations in column 4 between upper and lower halves in both tables). Contrary to the NH listeners, inter-subject variability is increased for the HI listeners (compare standard deviations in column 45 between upper and lower halves in both tables). As for the upper part of Table 1, a significant main effect of the listeners group (*p* < 0.05) on variance was found performing a two-way mixed-design ANOVA applied to Levene's absolute deviation estimate (column six in the row labeled with ANOVA). For the individual condition, significantly different variances were found for LNN with low to medium center frequencies (0.25, 0.5, 1, and 2 kHz) and aided IFN in Dataset 1. No significant differences are observed for LNN with high center frequencies (4 and 6 kHz) and unaided IFN. On the contrary, in Dataset 2 where more listeners participated in the measurements for broadband conditions (8 NH and 10 HI listeners in Dataset 2 compared with the subset of 4 NH and 4 HI listeners in Dataset 1), a significant difference is found for unaided IFN.

The mean loudness ratios are generally higher for the HI listeners than for the NH listeners for all conditions in both datasets; but no significant main effect of the listener group was found performing a two-way mixed-design ANOVA applied to Dataset 1.

Except for the IFN where only four listeners participated in the measurements for Dataset 1, comparable measurement conditions yielded similar results between datasets. The ratios of the NH groups are similar for both datasets for narrowband noises with a center frequency of approximately 1 kHz: The ratio for assumed equal loudness in ears inferred from Dataset 1 for the LNN stimulus with a center frequency of 1 kHz is 1.39 ± 0.3. The ratio for assumed equal loudness in ears inferred from Dataset 2 for the UEN1 stimulus (center frequency: 1.37 kHz) is 1.28 ± 0.16. The respective ratios for the HI groups are both higher than for the NH groups: the ratio is 1.97 ± 0.95 for Dataset 1 and 1.68 ± 0.69 for Dataset 2.

Binaural summation ratios derived from Dataset 2 suggest an increase in the ratio with bandwidth in the NH group, as the ratio for assumed equal loudness in the ears for the (unaided) IFN stimulus is higher (1.79 ± 0.35) than for the UEN1 stimulus (1.28 ± 0.16). On the contrary, ratios derived from Dataset 1 show only a slight increase for unaided IFN (1.49 ± 0.32 compared with 1.39 ± 0.3 for 1 kHz LNN) and no increase for aided IFN (1.27 ± 0.09). For the HI group of Dataset 1, the ratios for assumed equal loudness in the ears suggest increased binaural summation for aided IFN (2.69 ± 2.32 compared with 1.97 ± 0.95 for 1 kHz LNN) but a decrease for unaided IFN (1.55 ± 0.63). Ratios derived from Dataset 2 suggest an increase for both aided and unaided IFNs (3.65 ± 2.45 and 2.97 ± 1.27, respectively, compared with 1.68 ± 0.69 for aided UEN1).

Taken together, based on the binaural loudness summation data for narrowband stimuli, it was shown that for NH no level dependency and for HI no systematic level dependency of binaural summation exist. For both the NH and HI listeners, binaural summation ratios slightly decreased with frequency if averaged across listeners. Some of the HI listeners showed loudness ratios >2, i.e., indicating super additivity (binaural excitation). Individualization of the amount of binaural summation in the model can reduce the error in fitting the data by 10 CU (two loudness categories) in the high loudness region (or high stimulus levels). The binaural stage allows the calculation of “corrected” binaural summation ratios for assuming equal loudness, enabling better comparability between conditions, listeners, and other studies. The inter-subject variability of the “corrected” ratios is higher for the HI listeners than for the NH listeners. Mean ratios were higher in all the conditions for HI; however, the effect was not significant in most conditions, likely because of the small sample size.

### Experiment II: Individual Parameters to Describe Monaural and Binaural Loudness

**Figure 6:**
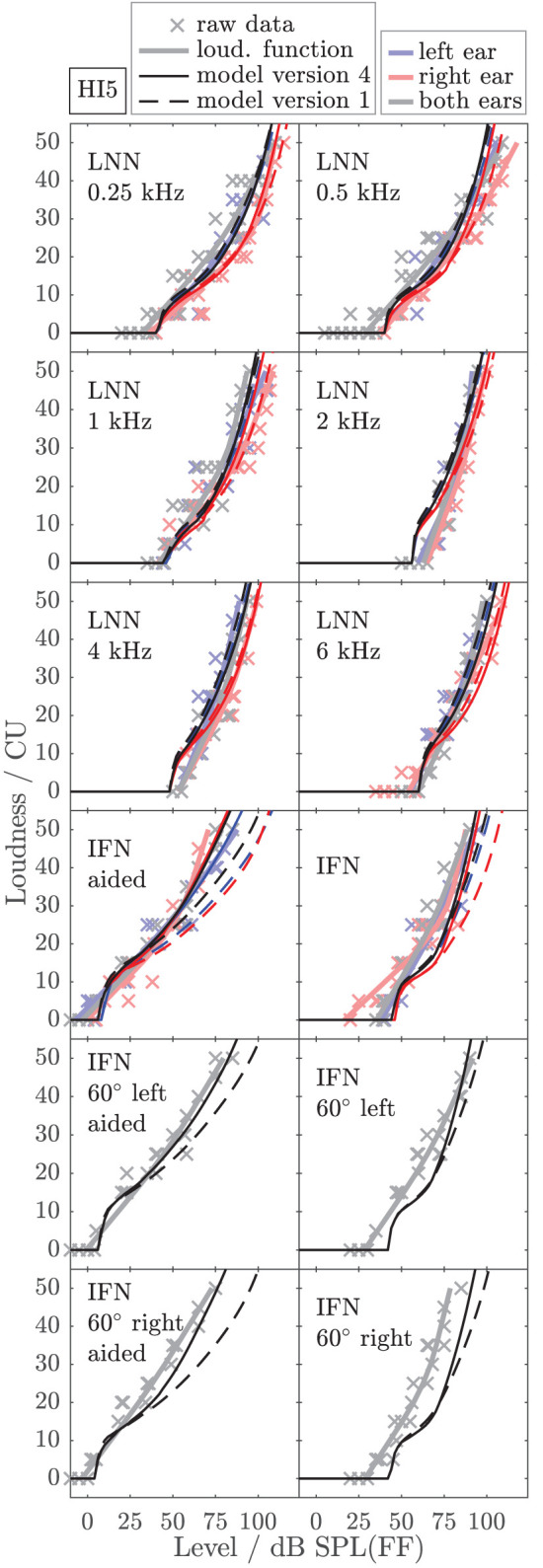
Empirical loudness functions of listener HI5 (brightened thick solid lines) and modeled loudness (thin lines) for monaural conditions (left ear: blue, right ear: red) and diotic conditions (gray/black) with simulated frontal sound incidence or from ±60° in the horizontal plane as indicated in the panels. The raw measurement data are indicated by crosses. Thin solid lines are for model version 4, for which all monaural (β_*L*_ and β_*R*_) and binaural parameters (α_*B*_ and β_*B*_) have been individualized by fitting the model to the LNN and the aided IFN stimuli. The loudness of four IFN stimuli in the lower panels and the unaided IFN are model predictions. Thin dashed lines are for model version 1, i.e., without bandwidth-dependent monaural and binaural gains (β_*L*_ = β_*R*_ = β_*B*_ = 0) and with average NH binaural inhibition (α_*B*_ = −0.273, see Appendix). For this model version, only the monaural LNN loudness data were used in the fitting procedure. The modeled loudness for the remaining binaural LNN stimuli and all IFN stimuli is a prediction.

**Figure 7:**
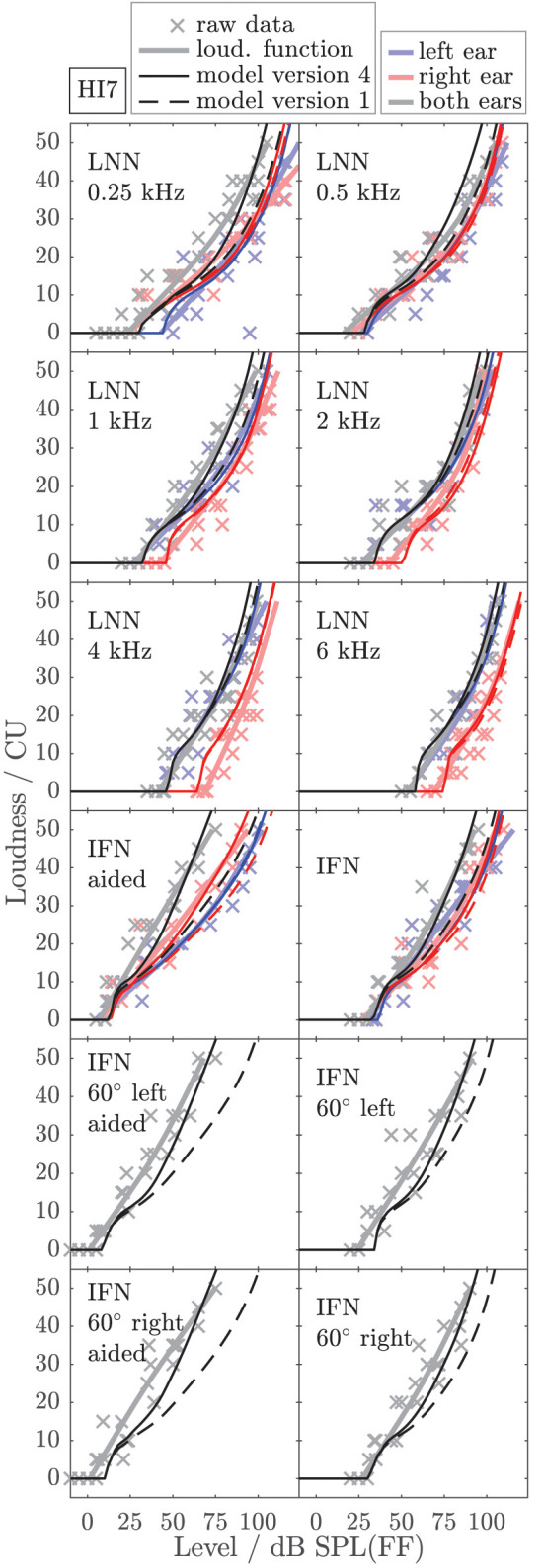
As Figure 6 but for listener HI7.

To exemplarily show the effects of individualized parameters for modeling loudness, Figures 6, 7 show the empirically derived loudness functions (thick solid lines, lightened colors), the underlying raw data (crosses), and modeled loudness functions (thin lines, darkened colors) of listeners HI5 and HI7. The corresponding figures for the subgroup of listeners for which broadband conditions were measured in Dataset 1 are provided in the Supplementary Material. The dashed lines show model version 1, for which only the monaural post gains have been individualized. The solid lines are for model version 4, for which all monaural (post gains, β_*L*_ and β_*R*_) and binaural parameters (α_*B*_ and β_*B*_) have been individualized. Red and blue indicate monaural presentation to the left and right ears, respectively. Gray and black indicate diotic presentation.

The model output of model version 1 (dashed lines) closely fits the loudness functions of the monaural LNN stimuli, which have been targeted in the fit (upper six panels, center frequencies indicated inside the panels). Model predictions for the monaural broadband stimuli are inaccurate (compare thin dashed red and blue lines with thick solid red and blue lines in the panels indicated as IFN and IFN aided). This result is in line with Pieper et al. (2018) who have shown that the fit of the post gain to narrowband loudness data does not improve the model predictions for broadband loudness data.

In model version 4 (solid lines), the aided monaural IFN stimuli were added to the targeted stimuli. Consequently, the modeled loudness functions better account for these data. However, the fit slightly alters the modeled loudness functions for the monaural narrowband LNN stimuli. Particularly at low frequencies, this can result in decreased accuracy of the model for narrowband stimuli (see, e.g., Figure 6, red lines in the panels indicated as 0.5 kHz and 1 kHz). Model version 4 improves the predictions for the unaided and aided monaural and binaural broadband stimuli, which were not involved in the fitting procedure (see panels indicated as IFN, IFN 60° left, IFN 60° left aided, IFN 60° right, and IFN 60° right aided in Figures 6, 7), which also holds for the other two HI listeners (not shown).

**Figure 8:**
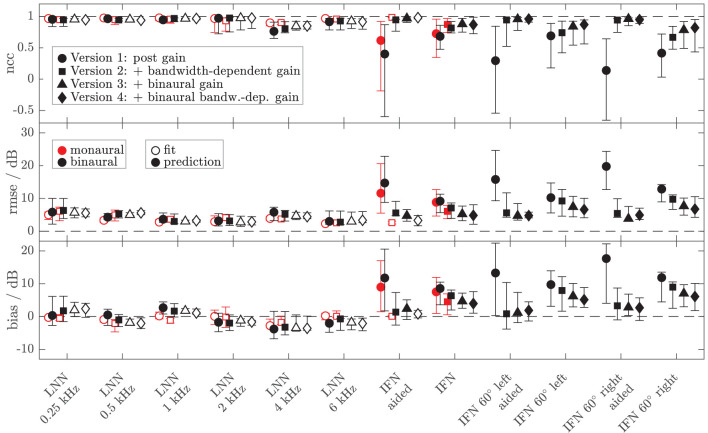
Median (symbols) and 25 and 75 percentiles (bars) of the model performance measures (top panel: ncc, middle panel: rmse, bottom panel: bias) for monaural (red, mean value across left and right ear per listener) and diotic (black) conditions across the subgroup of four HI listeners for which loudness data of broadband IFN stimuli were collected in Dataset 1. The dashed lines indicate optimal performance. Four different model versions were tested as described in the Method section of experiment II (circles: version 1 with monaural post gain, squares: version 2 with additional monaural bandwidth-dependent gain, i.e., individualized parameters β_*L*_ and β_*R*_, triangles: version 3 with additional overall binaural gain, i.e., individualized parameter α_*B*_, diamonds: version 4 with additional binaural-bandwidth-dependent gain, i.e., individualized parameter β_*B*_). Open symbols mark the conditions that were utilized in the model fits. Filled symbols indicate model predictions.

**Figure 9:**
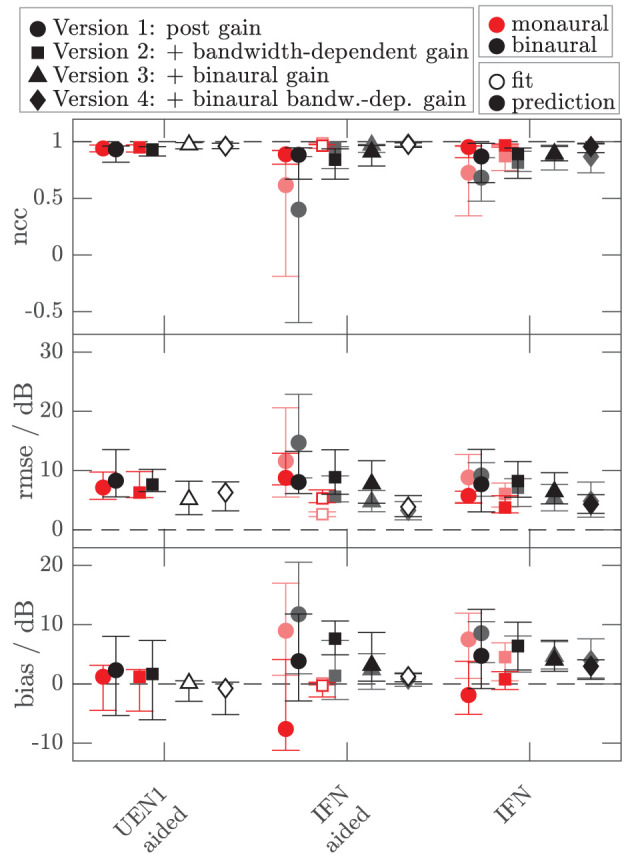
As Figure 8 but for the additional data of 10 HI listeners of Dataset 2. Performance measures from Figure 8 are replotted in brightened colors if measurement conditions are comparable. In contrast to Figure 8, the performance measures for the monaural narrowband LNN stimuli are not shown. Binaural or diotic data are not available for the LNN stimuli. Instead, the loudness data of the aided UEN1 stimulus were used to fit the parameters of the binaural stage.

To assess the performance of all the models and both data sets, Figures 8, 9 show the median (symbols) and the 25 and 75 percentiles (bars) of the performance measures (ncc, rmse, and bias) across the HI subgroup of Dataset 1 (Figure 8) and the HI listeners of Dataset 2 (Figure 9). While the figures show the performance measures for each stimulus in isolation, Table 3 lists the adjusted ncc' values across all the stimuli for the HI listeners, and Table 4 for the NH listeners.

**Table 3:** Adjusted ncc' (Equation 10) across all conditions.

**Model version**	**Number of parameters**	**Number of observations used in fit**	**25 percentile**	**Median**	**75 percentile**
**SUBGROUP OF DATASET 1 (4 LISTENERS, 280 OBSERVATIONS PER LISTENER)**					
1	36	120	0.765	0.840	0.904
2	38	140	0.919	0.936	0.949
3	39	200	0.937	0.944	0.956
4	40	210	0.935	0.952	0.963
3 modified	39	160	0.936	0.952	0.962
**DATASET 2 (10 LISTENERS, 210 OBSERVATIONS PER LISTENER)**					
1	36	120	0.901	0.933	0.958
2	38	140	0.905	0.950	0.960
3	39	150	0.947	0.959	0.964
4	40	160	0.946	0.964	0.971
3 modified	39	160	0.947	0.961	0.971

*Shown are the 25 percentiles (column 4), the medians (column 5) and the 75 percentiles (column 6) across the HI models for the different model versions (column 1). Column 2 shows the total number of parameters for each model version. Hearing loss, distribution of OHC/IHC loss, and post gain at 6 frequencies per ear yield a total of 36 parameters for model version 1. Model version 2 introduces one parameter per ear (bandwidth-dependent gain), version 3 introduces the binaural gain, and version 4 introduces the bandwidth-dependent binaural gain. Column 3 shows the number of observations used to determine the parameter values in the model fit. Observations are 10 loudness categories for each stimulus (0 CU lies below the absolute threshold and is therefore excluded). The number of stimuli and, therefore, the number of observations differ across datasets. The model version denoted “3 modified” uses the same (Dataset 2) or comparable (Dataset 1) stimuli in the model fit as model version 4 in Dataset 2*.

**Table 4:** Same as Table 3 but for NH listeners.

**Model version**	**Number of parameters**	**Number of observations used in fit**	**25 percentile**	**Median**	**75 percentile**
**SUBGROUP OF DATASET 1 (FOUR LISTENERS, 280 OBSERVATIONS PER LISTENER)**					
1	36	120	0.934	0.955	0.968
2	38	140	0.964	0.973	0.980
3	39	200	0.967	0.976	0.981
4	40	210	0.966	0.975	0.981
**DATASET 2 (EIGHT LISTENERS, 180 OBSERVATIONS PER LISTENER)**					
1	36	120	0.953	0.965	0.976
2	38	140	0.960	0.974	0.979
3	39	150	0.961	0.975	0.979
4	40	160	0.962	0.975	0.980

For the HI listeners of both datasets, the monaural bandwidth-dependent gain in model versions 2–4 (disabled in model version 1, β_*L*_ = β_*R*_ = 0) improves the model predictions across listeners for monaural broadband conditions (IFN unaided and aided, model version 1: red circles, model versions 2–4: red squares in Figures 8, 9). For the aided IFN stimuli, the rmse is reduced from 11.6 to 2.6 dB for the HI subgroup of Dataset 1 and from 8.8 to 5.3 dB for the HI group of Dataset 2. For the respective unaided IFN stimuli, which were not considered in the fits, the rmse is reduced from 8.8 to 6.1 dB for the HI subgroup of Dataset 1 and from 5.7 to 3.8 dB for the HI listeners of Dataset 2. Performance improvements are nearly as high for the respective NH listeners of both Datasets (not shown in figures). Median rmse values for aided IFN are reduced from 7.6 to 4 dB for the NH subgroup of Dataset 1. Median rmse values for unaided IFN are reduced from 6.8 to 5.5 dB for the NH subgroup of Dataset 1 and from 6.7 to 4.5 dB for the NH listeners of Dataset 2. The median of the adjusted ncc' values across the HI listeners is increased from 0.84 for model version 1 to 0.936 for model version 2 for the subgroup of Dataset 1 and from 0.933 to 0.95 for Dataset 2 (Table 3). The respective values for the subgroup of NH listeners in Dataset 1 are 0.955 and 0.973 for model versions 1 and 2, and 0.965 and 0.974 for Dataset 2. The higher values and the smaller benefit for Dataset 2 reflect that this dataset contains fewer broadband conditions than Dataset 1.

It has already been shown in experiment I that the individualization of the overall binaural gain α_*B*_ is necessary to describe the binaural data for certain listeners. In experiment II, benefits from individualized binaural summation are reflected in the performance measures for model version 3 in both datasets (black triangles in Figures 8, 9). Compared with the performance measures of model version 2 (without individualized binaural summation; black squares), individualization in model version 3 leads to a slight increase in the median nccs and a slight decrease in the median rmses for most conditions, indicating small overall improvements for the HI listeners. The reduced percentile ranges of the ncc and rmse measures for all the conditions indicate improvements in the model predictions for certain HI listeners. The median adjusted ncc' is slightly increased from 0.936 to 0.944 for the HI subgroup of Dataset 1 and from 0.950 to 0.959 for the HI listeners of Dataset 2. The respective 25 percentile is increased from 0.919 to 0.937 for the subgroup of Dataset 1 and increased from 0.905 to 0.947 for Dataset 2, indicating improvements in the worst-performing individual models. For the NH listeners, individualized predictions of model version 3 are almost not improved over model version 2.

The individualized model version 4 has been successfully fitted to the aided IFN without many tradeoffs for the narrowband stimuli (open diamonds in Figures 8, 9). However, the predictions of the HI data of Dataset 1 for all stimuli not used for the fitting procedure (closed diamonds) show no clear performance improvements in comparison with model version 3, whereas the predictions for the HI data of Dataset 2 are improved (unaided IFN only). For the HI group of Dataset 2, the median ncc for unaided IFN is increased from 0.89 for model version 3 to 0.96 for model version 4. The median rmse is reduced from 6.4 to 4.3 dB, and the median bias is reduced from 4 to 3 dB. The medians of the adjusted ncc' values are increased for both HI datasets (from 0.944 to 0.952 for the subgroup of Dataset 1 and from 0.959 to 0.964 for Dataset 2).

It has to be considered here that for version 4 the aided IFN data are added to the data used for fitting to allow a fit of the additional free parameter β_*B*_. However, nearly the same improvements can be archived when using model version 3 (i.e., no parameter β_*B*_) and including the same aided IFN data into the fitting procedure as well, referred to as model “version 3 modified.” The last row in the lower half of Table 3 shows the adjusted ncc' value of 0.961 for this modification for Dataset 2, which is almost as high as that for model version 4 (0.964). Likewise, the percentile values are comparable. The effect of this modification on the model performance was assessed for Dataset 1 as well. Again, aided IFN was added to the fitting data. Instead of the six binaural narrowband loudness functions originally used for fitting, only a single function (1 kHz) was used more comparable to the single UEN1 function (1.37 kHz) used for Dataset 2. Thus, comparable fitting data as for the above modified version 3 in Dataset 2 were used. Again, the resulting adjusted ncc' values are improved over the original model version 3 and are almost the same as those for model version 4 (see upper half in Table 3). Overall, although the additional parameter β_*B*_ improves the ability to fit the model to the loudness data, predictions of model version 4 are almost not improved over the predictions of model version 3 if the same underlying data are used for fitting. Using one binaural narrowband and one binaural broadband loudness function to determine α_*B*_ results in better performance than using the six binaural narrowband loudness functions.

The ncc' values only show a quite small increase for some of the models introducing binaural parameters. Given that the ncc' calculation includes more narrowband and monaural conditions than binaural and broadband conditions, differences between, e.g., models 2 and 3 might not be well-captured by the global ncc'.

**Figure 10:**
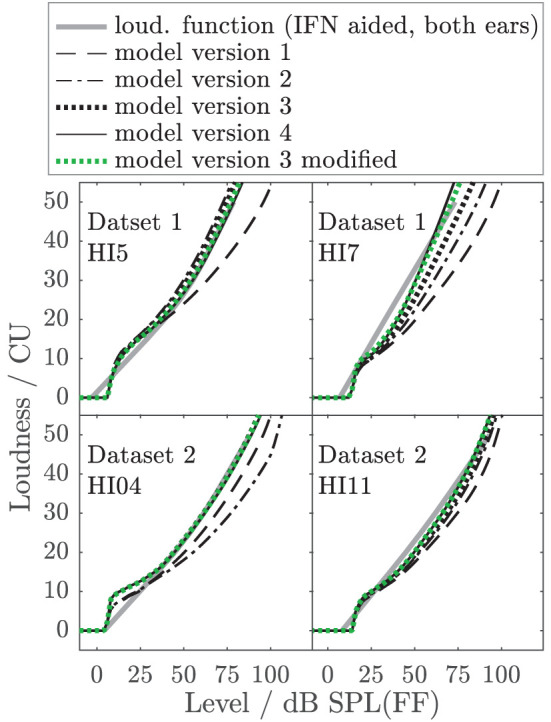
Examples of empirical loudness functions for aided binaural IF noise (solid gray lines) of listeners HI05 and HI07 of Dataset 1 (also shown in Figures 6, 7) and two listeners of Dataset 2 (HI04 and HI11), together with the respective predictions of all individualized model versions indicated in the legend.

To better illustrate the effect of the different model parameters, Figure 10 shows example aided binaural IF noise loudness functions (solid gray lines) of listeners HI5 and HI7 of Dataset 1 (also shown in Figures 6, 7) and two additional listeners of Dataset 2 (HI04 and HI11) together with the respective predictions of all individualized model versions. HI7 and HI04 are examples where model 2 (dash-dotted) considerably underestimates loudness by two categories (10 CU) at a level that is perceived as “too loud” (50 CU). Similar or worse mismatches are obtained for four more HI listeners (not shown). Model versions 3 (dotted black) and 4, (solid), and modified model version 3 (dotted green) considerably reduce the deviations for HI7 and HI04 and two other HI listeners. Two of 14 listeners remain with errors slightly higher than 10 CU (not shown). Thus, for realistic conditions with binaural speech-shaped noise, for six of 14 individuals model version 3 and higher avoid a severe underestimation of loudness in conditions that would otherwise lead to overly loud sensations, which are particularly problematic in the context of hearing aids. This demonstrates that even if the benefit of additional model parameters might be small on average, it can be highly relevant for individuals.

## Discussion

### Binaural Loudness Summation and Hearing Impairment

Both the modeling approaches in experiment II and the more data-driven approach in experiment I indicate decreased binaural inhibition (or even binaural excitation) in some of the HI listeners. This result appears to contradict the findings of van Beurden et al. (2018), who found similar binaural loudness summation between HI and NH listeners. The reason for this apparent contradiction is the differences in the methods on how to access and calculate binaural loudness summation. Whereas, in this study binaural loudness summation is calculated from the ratios between binaural and monaural loudness in sones at a given level (experiment I) or by fitting a single model parameter that alters the modeled binaural inhibition to the empirical loudness data (experiments I and II), van Beurden et al. (2018) calculated the level differences between monaural and binaural loudness functions at given loudness categories, but if the loudness ratios (in sones or CUs) are kept constant, the increase in the steepness of the loudness functions caused by the hearing impairment decreases the level differences between the functions (Moore et al., 2014). The fact that van Beurden et al. (2018) did not find such a decrease in these level differences in HI (in case of high hearing losses they even found a slight but not significant—increase) indicates that the loudness ratios at a given level must have been increased, which is in line with the observations in this study. On the contrary, Moore et al. (2014) found reduced level differences at frequencies where hearing loss was present. However, as mentioned in the introduction, a model that assumed average NH loudness ratios/inhibition predicted even lower-level differences and therefore underestimated binaural loudness summation in HI. To avoid conversion from CU to sones, other methods to directly assess loudness in sones, such as absolute magnitude estimation, appear suited. However, categorical loudness scaling has been shown to be well-applicable in the clinical context and for hearing aid fitting. Using absolute magnitude estimation, Marozeau and Florentine (2009) found increased inter-subject variability of the ratios in HI listeners, in line with the results of this study, but overall lower ratios and therefore no binaural excitation (*R* > 2). Their overall lower ratios can be explained with differences in the method: In this study, loudness in CU was transformed to loudness in sones using the transformation of Heeren et al. (2013). This transformation is based on the (sone-) loudness function in ANSI S3.4 (2007) resembling the loudness function in Hellman and Zwislocki (1961). Their loudness functions are considerably steeper than the respective loudness function derived with the method used by Marozeau and Florentine (see Epstein and Florentine, 2005). Rerunning the calculations in experiment I using the shallower loudness function yielded no binaural excitation, except for few stimuli for individual HI listeners. The increased inter-subject variability in the HI group, compared with that in the NH group, was still significant.

Based on this consideration, we hypothesize that the underlying physiological basis for *binaural excitation* (*R*>2) could be: (1) super-additivity: neural excitation from both sides is not added, but excitation from the contralateral side causes excess excitation on the ipsilateral side; and (2) an internal loudness representation with another slope than the sone scale used in this study in which case one would not observe *R* = 2 even if the binaural summation was purely additive.

Another limitation of this study might be the low sample size of eight NH and eight HI listeners for Dataset 1, and eight NH and 10 HI listeners for Dataset 2.

### Individualization of Loudness Models

It has been shown that for some HI individuals, severe deviations from average loudness perception exist, likely causing problems in daily life and with hearing aids (Oetting et al., 2018; van Beurden et al., 2020). Even if only a subgroup of HI listeners is affected, loudness models that aim to support hearing aid fitting and development need to account for these listeners. Current HI loudness models fail in this regard (Pieper et al., 2018).

In Pieper et al. (2018), a monaural frequency-dependent post gain was introduced. The post gain allows fitting of the loudness model to individual narrowband loudness data but does not improve the model predictions for broadband loudness. In experiment II of this study, a bandwidth-dependent gain has been added to the monaural paths, controlled *via* parameters β_*L*_, and β_*R*_. The monaural bandwidth-dependent gain improves the ability of the loudness model to describe and predict monaural broadband data for both the NH and HI listeners.

The results of experiments I and II show that accounting for individual increased binaural loudness summation can decrease prediction errors of binaural loudness for narrowband and broadband stimuli. This holds in particular for a subgroup of the HI listeners and is almost independent of frequency and bandwidth.

In order to allow for individual binaural loudness summation, a binaural gain has been introduced that is controlled *via* parameter α_*B*_. The binaural gain linearly attenuates (if α_*B*_ < 0) or amplifies (if α_*B*_ > 0) the signal, the more the signal amplitudes in the monaural paths are equal. Unlike the frequency-dependent post gain but like parameters β_*L*_ and β_*R*_, α_*B*_ is a single parameter, independent of BM location and therefore independent of stimulus frequency. If modeling average NH inhibition (by setting α_*B*_ to the average value of −0.273 of the NH listeners), the predictions of individual binaural loudness data were inaccurate for the HI listeners compared with the more accurate predictions for the NH listeners. Allowing individual values α_*B*_ for the NH listeners slightly improved the ability to fit the binaural narrowband data but did not improve the predictions of binaural broadband data. For the HI listeners, predictions were improved and similar prediction accuracies as for the NH listeners were obtained.

A mechanism similar to the monaural bandwidth-dependent gain has been added to the binaural path, controlled *via* parameter β_*B*_. The mechanism increases (for β_*B*_ > 0) or decreases (for β_*B*_ < 0) the binaural gain, the larger the bandwidths are and the more similar the signal amplitudes are in the monaural paths. This modification of the binaural gain did not further improve the predictions of the binaural broadband data, indicating that the individual amount of binaural inhibition would be almost independent of the bandwidth of the signal representation after monaural processing.

Therefore, it can be recommended that for the individualization of loudness models, the individual monaural spectral loudness summation should be addressed, independently of sensorineural hearing loss. If hearing loss is present, binaural loudness summation might be affected and therefore needs to be individualized as well. A single bandwidth-independent binaural gain (controlled here *via* parameter α_*B*_), i.e., model version 3, might be sufficient for most applications.

In this study, a subset of the individual loudness data has been used to determine the parameter values of an individual model. The “cost” for the measurement time to obtain this subset might be too high for certain applications, in particular for clinical use. Most time consuming are the 12 monaural narrowband loudness functions that were used to obtain the OHC loss, IHC loss, and post gain. Approximations of these functions could be derived from hearing thresholds and UCL measurements to reduce measurement time. Two monaural broadband loudness functions were used to determine the values of β_*L*_ and β_*R*_. Values of β_*L*_ and β_*R*_ were similar for most but not all listeners (not shown), so that the required measurement time cannot be reduced. Using one binaural narrowband loudness function and one binaural broadband loudness function to determine the individual value of α_*B*_ resulted in better model performance than using six binaural narrowband loudness functions.

Overall, to obtain a well-performing individual loudness model for an NH or HI listener, the measurement of 14 monaural loudness functions (12 narrowband, and two broadband) was required. For the HI listener, two additional binaural loudness functions (one narrowband, and one broadband) were required. Balancing “cost” and “value” of the measurements, a substantial reduction in measurement effort might be possible if the 12 monaural narrowband loudness functions are approximated based on more clinical data, such as the hearing threshold and uncomfortable level.

### Frequency Dependency of Binaural Summation

The results of experiment I suggest high individual but only slight systematic frequency dependencies of binaural loudness summation averaged across listeners. Comparing the averaged results from NH and HI listeners, increased binaural loudness summation was found for the HI listeners (see Tables 1, 2). This might suggest a connection between hearing loss and binaural loudness summation, which might also occur for frequency-dependent hearing loss within a listener. Given that hearing loss is typically increased at high frequencies, one could, thus, expect increased binaural summation at high frequencies. Contrary to this consideration, all except one HI listener (HI04) show decreasing summation ratios with increasing stimulus frequency (Figure 4). On average across listeners, decreasing summation ratios with increasing frequency was observed for both the NH and HI listeners. However, the binaural summation (parameter α_*B*_) of the model was chosen to be independent of frequency. Consequently, on average, the binaural loudness summation is underestimated at low frequencies (compare positive bias values for binaural conditions with bias values close to zero for monaural conditions for LNN stimuli with low center frequencies in Figure 8) and overestimated at high frequencies (compare negative bias values for binaural conditions with bias values close to zero for monaural conditions for LNN stimuli with high center frequencies in Figure 8) in the model calculations of experiment II. Such underestimation at low frequencies was already observed in Moore et al. (2014) using a model that also assumed binaural inhibition to be independent of frequency. Thus, further small improvements can be expected for the predictions of narrowband signals if a slight decrease in binaural summation with increasing frequency would be considered. This can be realized in this model by a decrease in the value of α_*B*_ as a function of the center frequency of the TLM segment.

### Relations to Hearing Impairment and Physiologic Mechanisms

In this model, the binaural stage is located subsequent to the simulation of basilar membrane movements and monaural central gain mechanisms (post gain and bandwidth-dependent central gain). Therefore, binaural inhibition is considered to be a more central effect. Before its introduction into binaural loudness models (Moore and Glasberg, 2007), the idea of central binaural inhibition mechanisms has been used in binaural auditory models for sound localization and binaural unmasking (Lindemann, 1986; Breebaart et al., 2001). Breebaart et al. (2001) argued that a subgroup of cells in the mammalian lateral superior olive and the inferior colliculus are excited by signals from one ear and inhibited by signals from the other ear. Since there is evidence for homeostatic adaptations of the neurons to reduced firing rates at the inputs in case of hearing impairment (Qiu et al., 2000; Kotak et al., 2005), reduced binaural inhibition might be a side effect of these adaptations. However, although central inhibition can explain the mentioned psychoacoustic observations, the link between neural stimulus encoding and the neural representations of percepts, including loudness, is not well-understood (Schreiner and Malone, 2015). Further candidates that can potentially influence binaural loudness summation are efferent reflexes like the MEM or the medial olivocochlear (MOC) reflex. The MOC reflex is directly affecting the cochlear gain (e.g., Berlin et al., 1993), whereas the MEM reflex causes a reduction of sound transmission by the middle ear of up to 10 dB for high stimulus levels at frequencies below 1 kHz (Rabinowitz, 1977). In both MEM reflex and MOC reflex, threshold and strength depend on stimulus level, frequency, and bandwidth as well as stimulus presentation (monaural or binaural). Both reflexes are feedback mechanisms that are controlled by post cochlear processes, and they are affected by damages to the auditory path prior to the central processing stages. For example, the characteristics of the MEM reflex threshold depend on the different peripheral compression in the NH and HI listeners (Müller-Wehlau et al., 2005). Thus, these effects might provide a direct link between the individual state of outer and inner hair cells and the individual amount of binaural inhibition. If the influence of the hair cell states on binaural inhibition is high in comparison to central binaural inhibition effects, a proper implementation of these feedback mechanisms could reduce the number of parameters that are required for the individualization of loudness models.

By reducing the cochlear gain of the TLM, the proposed loudness model accounts for reduced spectral loudness summation in the HI listeners. Nevertheless, subsequent to the TLM, significant bandwidth-dependent gain changes (controlled *via* parameters β_*L*_ and β_*R*_) were necessary to describe the individual monaural broadband data (results of experiment II). These subsequent corrections were not only necessary for the HI but for the NH listeners as well, for which only small cochlear gain losses were expected and therefore simulated. Together with the finding that a similar mechanism applied to the binaural path of the model did not improve binaural loudness predictions of broadband stimuli, the model simulations suggest an additional mechanism besides the cochlear non-linearities that influence spectral loudness summation, which is not related to hearing loss, as already hypothesized in Pieper et al. (2018).

### Implications for Loudness Models and Application in Hearing Aid Fitting and Development

This model analysis estimated the number and type of monaural and binaural parameters required to improve fitting to and prediction of individual loudness data. It is generally expected that an increasing number of free parameters increase the ability of any model to fit the data. The goal was, therefore, to systematically assess the benefit of successively adding perceptually motivated and physiologically plausible, effective model stages with respective parameters and to devise the minimum number required for individualization. We show that, in fact, additional stages beyond commonly considered peripheral processes, such as non-linear gain loss and linear attenuation, typically associated with OHC and IHC, loss are required to account for individual loudness data in NH and HI. At least a bandwidth-dependent retro-cochlear gain parameter, likely reflecting central gain, is required to individualize the amount of spectral summation, and a frequency- and bandwidth-independent binaural gain parameter is required to individualize binaural summation (inhibition or excitation). The authors deem it unlikely that individual loudness can be accounted for with any improved peripheral model without the need for the suggested or similar additional parameters.

Although a specific loudness model was used in this study, the findings can be generally applied to other loudness models and do not depend on the front end of the current model. Other loudness models (e.g., Chalupper and Fastl, 2002; Chen et al., 2011a; Moore et al., 2014) could be extended with the suggested or modified retro-cochlear stages, which introduce additional individual parameters. The front ends of these models offer the advantage of strongly reduced computational complexity compared with the current TLM front end.

The potential of individualized loudness predictions is in hearing aid fitting, where a fitting rule can contain the individualized model and improved hearing aid gains can be devised for different situations based on the respective prominent signal properties and the wearers individual loudness perception. Further potential is in hearing aid development, where loudness can be predicted for a certain set of prototypical HI with different loudness perceptions. Future potential can be expected in hearing aid algorithms with real-time updates of their processing based on integrated individual loudness predictions. Although there is still room for further improvement of individual loudness predictions, the relevance of the already achieved, at times seemingly small differences or improvements in dB, should not be underestimated. Due to the steep progression of HI loudness functions, in particular close to uncomfortable levels, according gain changes in hearing aids can easily make the difference between acceptable and uncomfortable loudness.

## Summary and Conclusions

Loudness perception of the NH and HI listeners with sensorineural hearing loss was measured by categorical loudness scaling for narrowband and broadband stimuli, presented monaurally, and binaurally. To assess the individual amount of binaural summation, binaural loudness ratios were calculated, and a data-driven model approach was employed to account for binaural loudness based on the measured monaural loudness by individually fitting a single binaural summation parameter, α_*B*_. Analysis of the loudness data showed a higher individual variability of binaural loudness summation for HI compared with the NH. While NH showed binaural inhibition in line with previous findings from the literature, the data of some of the HI listeners of this study suggest reduced binaural inhibition (α_*B*_ < 0) or even super additive summation, i.e., binaural excitation (α_*B*_ > 0).

In the second step, the monaural loudness model of Pieper et al. (2018) was extended by a functional binaural loudness summation stage (Equation 3). The stage sums the signals in the monaural paths and weights the result depending on the amplitude difference in the monaural paths and the value of the parameter α_*B*_ that controls the overall amount of binaural inhibition or excitation. Loudness model predictions for binaural stimulus presentation were improved for individual HI listeners if the individual amount of binaural inhibition/excitation was considered. The introduction of an additional parameter β_*B*_ that alters the amount of binaural inhibition/excitation depending on the bandwidth of the summed monaural signals did not substantially improve the model predictions. However, for the accuracy of the model predictions in both the NH and HI listeners, it was crucial to include bandwidth-dependent weightings of the signals in the monaural paths (Equation 1) that were controlled with parameters β_*L*_ and β_*R*_ for the left and right ears, respectively.

The following conclusions can be drawn:

1. Individual ratios of binaural loudness summation vary across the HI listeners and are sometimes increased compared with the NH listeners, indicating that binaural inhibition, as typically observed in NH, might be affected by sensorineural hearing loss.
2. The empirical data suggest a slight increase in binaural inhibition (or decrease in binaural excitation) with frequency for both the NH and HI listeners.
3. To correctly account for spectral loudness summation, individualized loudness models for NH and HI should include an individually adapted bandwidth-dependent retro cochlear gain stage in the monaural pathway.
4. Individualized loudness models for HI listeners should account for the individual amount of binaural inhibition/excitation.

## Data Availability Statement

The original contributions presented in the study are included in the article/Supplementary Material, further inquiries can be directed to the corresponding author/s.

## Ethics Statement

The studies involving human participants were reviewed and approved by the Ethics Committee of the University of Oldenburg. The patients/participants provided their written informed consent to participate in this study.

## Author Contributions

IP, MM, and SE co-conceived the presented ideas. SE supervised the project. IP developed the test software and carried out the simulations and experiments. All the authors discussed the results and contributed to the manuscript.

## Conflict of Interest

The authors declare that the research was conducted in the absence of any commercial or financial relationships that could be construed as a potential conflict of interest.

## Footnotes

^1^Listeners were the same as in Pieper et al. (2018). As in Pieper et al. (2018), a ninth NH listener was excluded from the original dataset because the listener's uncomfortable levels were identified as outliers at three of the six test frequencies.

^2^The extreme case where the signal is only perceived in one, for example the left, ear (*N*_*L*_ = *N*_*B*_ and *N*_*R*_ = 0) Equation 6 will result in a ratio *R* = 2.

^3^The aided broadband condition was chosen because pretests revealed better overall predictions than for using the unaided condition.

^4^Good convergence of the fits was achieved if the broadband IF noise data was weighted half as high as the narrowband LNN data and the weighting of the adjacent post gain differences was increased. Weighting of LNN = 1 (x6 signals), weighting of IFN = 3, weighting of post gain differences: 1 (Pieper et al., 2018: weighting of LNN = 1 (x6 signals), weighting of IFN = 0, weighting of post gain differences: 1/5).

^5^Biases may be possible due to the choice of the loudness function that is used to fit the raw CU data. The function used is based on the assumption that low CUs are linearly related to the stimuli levels (Brand and Hohmann, 2002). Another possible cause for biases may be small inaccuracies in the transformation from CU to sones.

## Supplementary Material

The loudness functions and the underlying raw loudness data of 8 NH and 8 HI listeners collected for the current study (Dataset 1) as well as model calculations are provided.

## Appendix

### Binaural Model Stage for Normal Hearing and Refinement of Loudness Transformations

To obtain loudness in sones *S*_*m*_ (at time step *m*), a power law with the exponent *B* is applied to the internal binaural loudness *I*_*m*_ and the result scaled with the factor *A* (Pieper et al., 2016, 2018):

$$\begin{array}{l} S_{m}=A\cdot I_{m}^{B} \end{array}$$

Loudness in CU can then be obtained using the five-parameter cubic function of Heeren et al. (2013):

$$\begin{array}{l} CU=a_{3}\mathrm{lg}{\left(S/sone+b\right)}^{3}+a_{2}\mathrm{lg}{\left(S/sone+b\right)}^{2}\\ \;+a_{1}\mathrm{lg}\left(S/sone+b\right)+c \end{array}$$

In Pieper et al. (2018) the parameters of the transformations have been fitted to averaged NH binaural (*A* and *B*) and monaural (*a*_1_, *a*_2_, *a*_3_ *b*, and *c*) narrowband loudness data at 1 kHz, resulting in the values: *A* = 2.1·10^6^, *B* = 0.768, *a*_1_ = 8.8, *a*_2_ = 3.02, *a*_3_ = 4.47, *b* = 0.092, and *c* = 13.3. However, to fit the transformation from sones to CU, binaural loudness in sones was, for simplicity, divided by 2. Given that the current binaural summation stage differs from this assumption, these parameters needed to be refined. Furthermore, a binaural summation stage for averaged NH data was required for model versions 1 and 2 to access the benefit of its individualization in model version 3 and 4. For this, an iterative procedure was performed involving the fitting procedure of the loudness transformations and the individualization procedure of model version 3:

In the first iteration step, the parameter α_*B*_ of the binaural summation stage (Equation 3) was set to a value that reflects average NH inhibition. The transformations from internal loudness to loudness in sones and from sones to CU were fitted to average NH loudness data from other studies as mentioned above. In the first iteration α_*B*_ was set to −0.36 as inferred from experiment I.

In the second iteration step, the individual model version 3 was adjusted for all NH listeners of Datasets 1 and 2 as described in the method section of experiment II, using the loudness transformations from the first iteration step. The mean across the resulting individual values of α_*B*_ was then used for the binaural summation stage in the first step of the next iteration.

The iteration was repeated until the average value of α_*B*_ did not change by more than 1% from the previous iteration (here four iterations were sufficient). The resulting average value is α_*B*_ = − 0.273. The resulting parameter values for the transformations are *A* = 1.44·10^6^, *B* = 0.795, *a*_1_ = 6.23, *a*_2_ = 2.22, *a*_3_ = 3.709, *b* = 0.053, and *c* = 12.1. These transformations were used for all model versions in experiment II.
